# Exploring Dietary Behavior Changes Due to the COVID-19 Confinement in Colombia: A National and Regional Survey Study

**DOI:** 10.3389/fnut.2021.644800

**Published:** 2021-04-12

**Authors:** Sonia L. Pertuz-Cruz, Esther Molina-Montes, Celia Rodríguez-Pérez, Eduardo J. Guerra-Hernández, Olga P. Cobos de Rangel, Reyes Artacho, Vito Verardo, María Dolores Ruiz-Lopez, Belén García-Villanova

**Affiliations:** ^1^Department of Nutrition and Food Science, University of Granada, Granada, Spain; ^2^Departamento de Nutrición Humana, Facultad de Medicina, Universidad Nacional de Colombia, Bogotá, Colombia; ^3^Biomedical Research Center, Institute of Nutrition and Food Technology (INYTA) “José Mataix”, University of Granada, Granada, Spain; ^4^Instituto de Investigación Biosanitaria de Granada (ibs.GRANADA), Granada, Spain; ^5^Centro Investigación Biomédica en Red de Epidemiología y Salud Pública (CIBERESP), Madrid, Spain; ^6^Department of Nutrition and Food Science, University of Granada, Melilla, Spain

**Keywords:** dietary patterns, dietary habits, nutritional survey, culinary processes, COVID-19 confinement

## Abstract

The aim of this study was to evaluate the impact of coronavirus SARS-Cov2 (COVID-19) confinement measures in Colombia on the dietary behaviors of a large population sample, at national and regional levels. A survey was conducted to assess dietary behaviors during the COVID-19 confinement. The survey involved 2,745 participants, aged 18 years or older, from six regions of the country (Atlántica, Bogotá, Central, Oriental, Orinoquía and Amazonía, and Pacífica). Dietary intake of foods and foods groups in grams per day before and during the confinement was estimated by considering standard serving sizes of foods. One-way ANOVA was used to analyze differences between the regions with regard to dietary behavior changes during the confinement. Differences were deemed significant at *p-*value < 0.05. Dietary patterns (DPs) before and during the confinement were derived from principal component analysis. Certain dietary habits were adopted by the study population during the confinement (e.g., higher frequency of snacking and home cooking), with significant differences by regions with regard to these habits, as well as regarding culinary processes. The levels of consumption of several foods also changed during the confinement, nationally and regionally. We identified three DPs before the confinement (protein-rich, carbohydrate-rich, and sugar foods patterns) and four DPs during the confinement (westernized, carbohydrate-rich, protein-rich, fish and fruits-vegetable patterns), with an explained total variance of 33 and 45%, respectively. The profile of these DPs varied to some extent between the regions; their adherence to each DP also varied (*p*-value < 0.001). Our results show that there were marked differences by regions in the dietary behaviors of this population during the confinement, with an overall trend toward unhealthier DPs. These results may help to shape public health nutrition interventions in Colombia during the COVID-19 pandemic and in a post-COVID stage.

## Introduction

Public health measures implemented to halt the spread of coronavirus SARS-Cov2 (COVID-19) have produced major transformations in the society on a worldwide basis, both of qualitative and quantitative nature. For example, community confinement as a preventive isolation measure has been adopted by most countries to control the growth of the disease transmission, but this measure has been recognized as disturbing and problematic. One of its main social consequences is related to dietary behavior changes, which seem to have turned to healthier dietary patterns (DPs) in some European and Asian case studies (1). In Latin America, such confinement measures may have also changed dietary behaviors (2), but few studies have appraised the extent to which these measures have influenced the above. So far, a small study conducted among adolescents from Chile, Colombia, and Brazil noted an increased consumption of fried food, sweet food, legumes, vegetables, and fruits (3). A study from Chile, also reported major dietary changes in the population during the confinement (4). However, there is no study evaluating changes in dietary behaviors in the adult population from Colombia under this situation.

The Colombian government declared a mandatory confinement on March 25, 2020 (5), which remained in force, in full or in part by de-escalating means, until recently. This situation has given rise to changes in the daily life of the country's inhabitants, affecting not only social relations but also causing an economic disruption (6, 7). There have also been alterations in the food supply and consumption habits of the population; this being supported by national data on food demand and household expenditure. Detailed figures in this regard, with special emphasis on the population's dietary behaviors, are urgently needed to evaluate the impact of the COVID-19 confinement on this issue.

Prior to the COVID-19 pandemic, the Colombian National Survey on the nutritional status of the country of 2015 reported a significant growth in the indicators of obesity and overweight for the adult population between 18 and 64 years, reaching a 56.4% prevalence of excess weight (8). The adoption of unhealthier DP and lifestyle habits appear to be the main cause. An increased consumption of processed foods has been witnessed since the 1990s in this country, along with physical inactivity, longer working hours, and reduced mobility (8–10). The National Nutritional Surveys (ENSIN 2005 and 2015) also support the loss of traditional lifestyle and dietary habits (11). These changes have been associated with a higher prevalence of chronic non-communicable diseases such as cardiovascular diseases (12, 13) which are the main causes of mortality and morbidity in Colombia (14).

Besides, it is important to highlight that there are regional differences with regard to the aforementioned data on obesity and overweight, with Pacífica and Amazonía exhibiting the highest prevalence rates (8). The same trend has been made visible for DPs (11). Colombia is a country of great diversity relative to social and production systems, which affects food availability and supply (15). The current COVID-19 pandemic is supposed to affect the dietary behaviors in all regions of the country (16). It is therefore deemed necessary to characterize the changes in these habits of the Colombian population and to identify improvement needs in diet toward a better health and well-being.

This study aimed to assess how the COVID-19 confinement has influenced dietary behaviors of the Colombia adult population at the national and regional levels, with regard to the consumption of foods, the culinary processes applied, food safety measures, and other related variables. Herein, we describe for the first time dietary behaviors adopted by a large subset of the adult Colombian population taking survey data on changes in these behaviors since the adoption of the COVID-19 confinement.

## Methods

### Study Design and Population

A cross-sectional study with the administration of a survey on dietary behaviors during the COVID-19 confinement was carried out. There were 2,745 adults (aged over 18 years) from Colombia participating in the survey. The survey was disseminated using snowball sampling strategies all over Colombia through different channels (mailings, conferences, social media, and press releases) and was running from the second to the eighth week of confinement.

The study protocol was approved by the Human Research Ethics Committee of the University of Granada (1526/CEIH/2020) since this study is part of the COVIDiet-Int study (ClinicalTrials.gov number NCT 04449731). The survey (open between April 6 and May 22, 2020) was administered online, and all participants were informed about the objective of the research. All agreed to participate anonymously in the study and authorized the use of the data for research and dissemination. Also, the survey was conducted in agreement with the Declaration of Helsinki and in due compliance with personal data protection regulations in Colombia (Legislative Decrees 1581/2012 and 1377/2013). No further IRB approvals were requested.

### Data Collection

The survey was based on the one applied in the Spanish COVIDiet Study (17) and was further modified to adapt the questionnaire to the dietary behaviors of the Colombian population. Therefore, the same COVIDiet study survey was applied except for the dietary section on intake of foods. Given that, the dietary section did not account for Mediterranean Diet items as the Spanish study did, but for other foods typically consumed in Colombia. A pilot study was conducted among 20 volunteers to ensure the survey process and understanding and its effective implementation.

The questionnaire comprised 73 items within five distinct sections: (i) baseline characteristics (gender, age, type of housing, children in care, educational level, health status, height, and weight); (ii) dietary intake of foods during the confinement; (iii) cooking processes (cooking, frequency of frying and other culinary processes, type of oil used, and oil reutilization); (iv) lifestyle and dietary habits (physical activity, snacking, fast food consumption, meals out of home, water intake); (v) food security and safety issues (food hygienization, food availability and supply, expenditure on food, and staid aid). The final four sections referred to dietary behaviors. Furthermore, the participants were asked whether or not they changed their dietary behaviors during the confinement with regard to their usual practices. In addition, data on changes during the confinement in alcohol consumption and perception about weight gain was collected.

### Data Processing

Information on dietary intake regarded frequency of consumption in servings per day or per week (rating scale 0–5). Dietary intake was collected for 18 food groups: (1) cereals, (2) bakery and pastry, (3) tubers and plantains, (4) fruits and vegetables, (5) milk and dairy products, (6) red meat and processed, (7) poultry and processed, (8) fish, (9) eggs, (10) legumes, (11) fats (butter and margarine), (12) soft beverages, (13) sugar cane beverages, (14) coffee, (15) sugar or sugar cane, (16) desserts and sweets (desserts and ice cream), (17) snacks, and (18) nuts.

Moreover, since participants provided information on changes in the intake during the COVID-19 confinement, i.e., whether it was maintained the same, or whether it increased or decreased since the confinement was activated, it was possible to estimate the dietary intake of these foods before the confinement. In particular, we considered the reported intakes of food groups in servings/day. Then, this number of servings was kept the same when the dietary intake was reported to remain unchanged during the confinement. Instead, the number of servings was reduced by one when the intake increased during the confinement or was otherwise increased by one serving. In this way, we estimated the average servings of foods consumed by the participants before and during the confinement. Standard serving sizes in grams/day adapted from the Colombian dietary guidelines (18) were considered in order to estimate the dietary intake of these foods in grams/day (servings/day^*^grams/day of each serving), before and during the confinement. For cereals and legumes, a cooking factor was applied since their consumption was reported for cooked servings of these foods; weighted cooked servings were considered to quantify more precisely their intake. Thus, dietary intake information was obtained in servings/day and grams/day, before and during the confinement.

Finally, we estimated the week the survey was completed as a proxy of weeks of confinement by considering the difference in the launching and completion dates. Also, body mass index (BMI) was calculated by means of the reported weight and height of the participants [weight in kg/(height in m)^2^]. BMI categories as defined by the WHO were considered (19).

### Statistical Data Analysis

Descriptive statistics were derived for quantitative (mean and standard deviations) and categorical variables (frequencies and percentages). Relationships of variables by regions were also plotted using bar plots. Differences by regions were evaluated *via* one-way ANOVA or chi-squared test (or Fisher's exact-test for <5 observations), respectively.

Principal component analysis (PCA) was applied to obtain dietary clusters or DPs, i.e., components, before and during the COVID-19 confinement. Sixteen dietary items were used after collapsing some food groups: cereals including cereals and bakery and pastries; snacks including snacks and nuts. The components were rotated by orthogonal transformation (Varimax rotation) to facilitate their interpretation. Kaiser-Meyer-Olkin measure of sampling adequacy (KMO) and Bartlett's-test of sphericity values were >0.7, validating the use of PCAs in the study sample.

Eigenvalues >0.1 and scree plot graphs of the eigenvalue against the component number were considered to choose the optimum number of components. Then, factor loadings were obtained for each food group, to identify the groups most highly correlated with the DP. Food groups with factor loadings >0.30 were retained. The variability (variance) of the data explained by each component (pattern) was also calculated overall and by DP. Radial charts for visualizing and exploring DPs were used on the raw data (average serving sizes) and on the identified patterns.

Principal component analysis was applied on the entire study population due to sample size issues, although PCAs by regions were analyzed further to complement the analyses. Differences in adherence to the components by regions were evaluated to identify potential regional variations. For each DP, each subject received a score that was calculated as the sum of the intakes in each food group weighted by the corresponding factor loading. A higher score indicated a higher adherence to the respective DP. Non-parametric ANOVA-tests were considered (Kruskal-Wallis-test) to reveal differences by regions owing to the non-normal distribution of the scores (Kolmogorov-Smirnov-test, *p*-value < 0.05).

Likewise, all analyses were performed by weeks of confinement, to compare the short- and long-term impact of the confinement, i.e., the first 2 weeks (up to the fourth of confinement) with regard to the last 4 weeks (up to the eighth week of confinement), respectively.

Statistical significance was set at *p*-value < 0.05. The analyses were carried out with SPSS version 27 IBM for Windows (20), and R version 4.0.2 (21).

## Results

### Sociodemographic Characteristics of the Study Sample, Nationally, and by Regions

Table 1 shows sociodemographic characteristics of the respondents by regions. Among the six regions, there were significant differences (*p*-value < 0.05) in the proportion of men and women, number of children in the household, and educational attainment. A major proportion of respondents were women (73.1%) and University graduates (77%). The state of health requiring to follow a special diet and age groups also differed significantly between the regions. For instance, while the majority of participants were aged <51 years, there were small variations by regions in the distribution of age groups. Most respondents in Orinoquía and Amazonas were young-aged (72.5% of years 18–35), whereas fewer were aged older than 51 years in comparison with the other regions. The prevalence of participants with medical conditions varied from 11% (Orinoquía and Amazonas) to 26% (Atlántica). In addition, there were significant differences in weight (*p-*value = 0.013) and BMI (*p*-value < 0.001) between the regions, with Central and Bogotá regions showing the lowest obesity prevalence rates (<8%). There were no differences with regard to place of residence, state aid, support, and height.

**Table 1:** Baseline characteristics of the diet-COVID-19 survey respondents in Columbia by regions.

	**National**	**Atlántica**	**Bogotá**	**Central**	**Oriental**	**Orinoquía and Amazonas**	**Pacífica**	***p*-value^**a**^**
	***N* = 2,745 (%)**	***N* = 262 (%)**	***N* = 1,374 (%)**	***N* = 272 (%)**	***N* = 476 (%)**	***N* = 91 (%)**	***N* = 270 (%)**	
**Gender**^**b**^								0.013
Men	735 (26.8)	54 (20.6)	385 (28)	83 (30.5)	118 (24.8)	23 (25.3)	72 (26.7)	
Women	2,006 (73.1)	208 (79.4)	985 (71.7)	189 (69.5)	358 (75.2)	68 (74.7)	198 (73.3)	
**Place of residence**^**c**^								0.335
House	1,208 (44.0)	156 (59.5)	448 (32.6)	127 (46.7)	262 (55)	59 (64.8)	156 (57.8)	
Apartment	1,474 (53.7)	100 (38.2)	902 (65.6)	138 (50.7)	203 (42.6)	26 (28.6)	105 (38.9)	
Room	43 (1.6)	4 (1.5)	19 (1.4)	5 (1.8)	5 (1.1)	5 (5.5)	5 (1.9)	
Indigen home	20 (0.7)	2 (0.8)	5 (0.4)	7 (0.7)	6 (1.3)	1 (1.1)	4 (1.5)	
**Children in care**								0.006
0	1,647 (60)	145 (55.3)	862 (62.7)	172 (63.2)	277 (58.2)	39 (42.9)	152 (56.3)	
1	656 (23.9)	62 (23.7)	320 (23.3)	59 (21.7)	111 (23.3)	29 (31.9)	75 (27.8)	
2	358 (13)	44 (16.8)	158 (11.5)	35 (12.9)	71 (14.9)	20 (22)	30 (11.1)	
3	84 (3.1)	11 (4.2)	34 (2.5)	6 (2.2)	17 (3.6)	3 (3.3)	13 (4.8)	
**Educational level**								<0.001
None	10 (0.4)	1 (0.4)	4 (0.3)	1 (0.4)	0 (0)	1 (1.1)	3 (1.1)	
Primary school	33 (1.2)	3 (1.1)	10 (0.7)	10 (3.7)	4 (0.8)	1 (1.1)	5 (1.9)	
High school	326 (11.9)	22 (8.4)	161 (11.7)	29 (10.7)	47 (9.9)	20 (22)	47 (17.4)	
Graduate	263 (9.6)	20 (7.6)	142 (10.3)	14 (5.1)	45 (9.5)	9 (9.9)	33 (12.2)	
Professional	1,194 (43.5)	150 (57.3)	540 (39.3)	120 (44.1)	214 (45)	41 (45.1)	129 (47.8)	
Post-graduate	919 (33.5)	66 (25.2)	517 (37.6)	98 (36)	166 (34.9)	19 (20.9)	53 (19.6)	
**State aid**								0.594
No	2,625 (95.6)	241 (92)	1,336 (97.2)	257 (94.5)	462 (97.1)	79 (86.8)	250 (92.6)	
Yes	120 (4.4)	21 (8)	38 (2.8)	15 (5.5)	14 (2.9)	12 (13.2)	20 (7.4)	
**Age (years)**								<0.001
18–35	1,383 (50.4)	151 (57.6)	658 (47.9)	128 (47.1)	210 (44.1)	66 (72.5)	170 (63)	
36–50	791 (28.8)	66 (25.2)	408 (29.7)	81 (29.8)	159 (33.4)	18 (19.8)	59 (21.9)	
51–66	494 (18)	36 (13.7)	267 (19.4)	51 (18.8)	96 (20.2)	7 (7.7)	37 (13.7)	
>66	77 (2.8)	9 (3.4)	41 (3)	12 (4.4)	11 (2.3)	0 (0)	4 (1.5)	
**Health status**								0.004
No	2,237 (81.5)	193 (73.7)	1,125 (81.9)	227 (83.5)	382 (80.3)	81 (89)	229 (84.8)	
Yes	508 (18.5)	69 (26.3)	249 (18.1)	45 (16.5)	94 (19.7)	10 (11)	41 (15.2)	
**Weight**	66.1 ± 12.8	68.2 ± 14.8	65.4 ± 12.3	65.8 ± 12.5	67.1 ± 13.2	66.4 ± 13.4	66.0 ± 12.2	0.013
**Height**	1.64 ± 0.09	1.63 ± 0.09	1.64 ± 0.09	1.64 ± 0.08	1.63 ± 0.08	1.62 ± 0.08	1.65 ± 0.09	0.179
**BMI (kg/m)**								<0.001
<18.5	58 (2.3)	12 (5.1)	21 (1.7)	4 (1.6)	8 (1.8)	2 (2.4)	11 (4.5)	
18.5–24.9	1,467 (58.1)	107 (45.5)	778 (61.4)	164 (65.1)	232(52.3)	42 (51.2)	144 (58.8)	
25–29.9	770 (30.5)	84 (35.7)	372 (29.4)	67 (26.6)	152 (34.2)	27 (32.9)	68 (27.8)	
>30	230 (9.1)	32 (13.6)	96 (7.6)	17 (6.7)	52 (11.7)	11 (13.4)	22 (9.0)	

a Differences between groups were evaluated by chi-squared-test or one-way ANOVA where appropriate. Differences between groups were evaluated by Fisher's exact-test with <5 observations in some categories.

b Only four respondents from Bogotá identify themselves with another unspecified gender identity.

c *One respondent indicated other options*.

### Dietary Behaviors at National and Region Levels

Regional variations in the rates of consumers and non-consumers of specific food groups are shown in Table 2. Noteworthy, regardless of the region, over 90% of respondents consumed cereals, bakery, and pastry (except in the Pacifica region), tubers and plantains, fruits and vegetables (except in the Orinoquía and Amazonas regions), red meat and processed (except in the Pacifica, Central, and Bogotá regions), poultry, eggs, legumes, and nuts. Milk and dairy products were consumed by around 85% of the respondents, and coffee by 80% of them (except in the Orinoquía and Amazonas region for both food groups). Foods with consumption rates ranging between 50 and 75% were fish, fats, sugar or sugar cane, and sugar cane beverages (except in Central and Bogotá regions), and desserts and sweets (except in Atlántica and Orinoquía and Amazonas regions). Finally, consumption rates were lower (below 50%) for soft beverages (except in the Orinoquía and Amazonas region), snacks (40%) and alcoholic beverages (47%).

**Table 2:** Food groups consumed (C) and non-consumed (NC) by the diet-COVID-19 survey respondents in Colombia by regions.

**Food groups**	**National**	**Atlántica**	**Bogotá**	**Central**	**Oriental**	**Orinoquía and Amazonas**	**Pacífica**
	***N* = 2,745**	***N* = 262**	***N* = 1,374**	***N* = 272**	***N* = 476**	***N* = 91**	***N* = 270**
	**C/NC (%)**	**C/NC (%)**	**C/NC (%)**	**C/NC (%)**	**C/NC (%)**	**C/NC (%)**	**C/NC (%)**
Cereals	98/2	98/2	98/2	99/1	99/1	99/1	98/2
Bakery and pastries	91/9	88/12	92/8	94/6	98/10	92/8	86/14
Tubers and plantains	91/9	95/5	90/10	94/6	93/7	92/8	91/9
Fruits and vegetables	95/5	92/8	97/3	95/5	94/6	85/15	96/4
Milk and dairy products	85/15	85/15	86/14	83/17	83/17	71/29	87/13
Red meat and processed	89/11	91/9	89/11	88/12	93/7	91/9	86/14
Fish	71/29	77/23	74/26	67/33	70/30	64/36	64/36
Poultry and processed	96/4	95/5	96/4	93/7	97/3	92/8	95/5
Eggs	98/2	96/4	98/2	95/5	99/1	98/2	97/3
Legumes	93/7	93/7	92/8	95/5	92/8	93/7	96/4
Nuts	95/5	95/5	95/5	94/6	95/5	97/3	95/5
Fats	69/31	71/29	66/34	78/22	70/30	73/27	69/31
Soft beverages	36/64	46/54	33/67	35/65	36/64	51/49	36/64
Coffee	78/22	73/27	79/21	80/20	80/20	73/27	78/22
Sugar cane beverages	52/48	52/48	48/52	52/48	57/43	64/36	53/47
Sugar or sugar cane	61/39	73/27	57/43	56/44	64/36	75/25	63/37
Desserts and sweets	54/46	44/56	56/44	60/40	51/49	45/55	51/49
Snacks	40/60	40/60	41/59	36/64	34/64	44/56	39/61
Alcohol beverages	47/53	42/58	49/51	51/49	44/56	51/49	41/59

Table 3 presents changes in dietary behaviors due to the COVID-19 confinement by regions. Compared to dietary behaviors before the confinement, a higher frequency of snacking between meals was reported by nearly half of the respondents, or even more (63% in Orinoquía and Amazonas), but in the Central region (39%), the differences between the regions being statistically significant (*p*-value = 0.01). A higher consumption of fast food during the confinement was also reported in Orinoquía and Amazonas (36%) when compared to the other regions (<25%) (*p*-value = 0.005). Interestingly, Orinoquía and Amazonas respondents seemed to be more likely to gain weight (*p*-value = 0.003) despite reporting to practice more physical activity (*p*-value = 0.001) and home-cooking (*p*-value = 0.001) during the confinement when compared to the other regions. Respondents from the Orinoquía and Amazonas region were also found to eat more frequently out of home (before the confinement) (*p*-value = 0.032) and to experience difficulties in finding specific foods during the confinement compared to other regions (*p*-value < 0.001). Statistically significant differences (*p*-value < 0.05) between the regions were also observed for alcohol intake (higher in the Central region), water intake (higher in the Atlántica and Pacífica regions) and expenditure on food (higher in the Orinoquía and Amazonas region). However, no significant differences by regions were seen with regard to hygiene measures, perishable foods consumption and eating more at each meal.

**Table 3:** Dietary behaviors of the diet-COVID-19 survey respondents in Colombia by regions.

	**National**	**Atlántica**	**Bogotá**	**Central**	**Oriental**	**Orinoquía and Amazonas**	**Pacífica**	***p-*value^**a**^**
	***N* = 2,745 (%)**	***N* = 262 (%)**	***N* = 1,374 (%)**	***N* = 272 (%)**	***N* = 476 (%)**	***N* = 91 (%)**	***N* = 270 (%)**	
**Snacking**								0.011
As before	835 (30.4)	82 (31.3)	412 (30)	97 (35.7)	154 (32.4)	20 (22)	70 (25.9)	
Lower	593 (21.6)	62 (23.7)	299 (21.8)	69 (25.4)	90 (18.9)	14 (15.4)	59 (21.9)	
Higher	1,317 (48)	118 (45)	663 (48.3)	106 (39)	232 (48.7)	57 (62.6)	141 (52.2)	
**Fast food**								0.005
As before	1,238 (45.1)	106 (40.5)	621 (45.2)	134 (49.3)	226 (47.5)	31 (34.1)	120 (44.4)	
Lower	929 (33.8)	95 (36.3)	471 (34.3)	89 (32.7)	166 (34.9)	27 (29.7)	81 (30)	
Higher	578 (21.1)	61 (23.3)	282 (20.5)	49 (18)	84 (17.6)	33 (36.3)	69 (25.6)	
**Eat more**								0.275
As before	967 (35.2)	101 (38.5)	474 (34.5)	108 (39.7)	165 (34.7)	30 (33)	89 (33)	
Lower	544 (19.8)	43 (16.4)	287 (20.9)	61 (22.4)	87 (18.3)	16 (17.6)	50 (18.5)	
Higher	1,234 (45)	118 (45)	613 (44.6)	103 (37.9)	224 (47.1)	45 (49.5)	131 (48.5)	
**Physical activity**								0.001
Never	307 (11.2)	49 (18.7)	126 (9.2)	32 (11.8)	55 (11.6)	15 (16.5)	60 (22.2)	
As before	499 (18.2)	48 (18.3)	246 (17.9)	52 (19.1)	78 (16.4)	14 (15.4)	68 (25.2)	
Lower	1,317 (48)	124 (47.3)	672 (48.9)	127 (46.7)	235 (49.4)	17 (18.7)	28 (10.4)	
Higher	622 (22.7)	41 (15.6)	330 (24)	61 (22.4)	108 (22.7)	45 (49.5)	114 (42.2)	
**Weight gain**								0.003
No	1,036 (37.7)	89 (34)	539 (39.2)	113 (41.5)	174 (36.6)	22 (24.2)	99 (36.7)	
Yes	613 (22.3)	76 (29)	295 (21.5)	42 (15.4)	117 (24.6)	29 (31.9)	54 (20)	
Unknown	1,096 (39.9)	97 (37)	540 (39.3)	117 (43)	185 (38.9)	40 (44)	117 (43.3)	
**Meals out of home**^**b**^								0.032
Never	368 (13.4)	36 (13.7)	161 (11.7)	46 (16.9)	84 (17.6)	9 (9.9)	32 (11.9)	
1	513 (18.7)	48 (18.3)	280 (20.4)	37 (13.6)	83 (17.4)	13 (14.3)	52 (19.3)	
2	471 (17.2)	44 (16.8)	246 (17.9)	40 (14.7)	75 (15.8)	22 (24.2)	44 (16.3)	
3	1,393 (50.7)	134 (51.1)	687 (50)	149 (54.8)	234 (49.2)	47 (51.6)	142 (52.6)	
**Alcohol intake**								0.001
Never	1,445 (52.6)	151 (57.6)	694 (50.5)	133 (48.9)	265 (55.7)	45 (49.5)	157 (58.1)	
As before	607 (22.1)	44 (16.8)	336 (24.5)	68 (25)	97 (20.4)	16 (17.6)	46 (17)	
Lower	498 (18.1)	48 (18.3)	238 (17.3)	41 (15.1)	94 (19.7)	25 (27.5)	52 (19.3)	
Higher	195 (7.1)	19 (7.3)	106 (7.7)	30 (11)	20 (4.2)	5 (5.5)	15 (5.6)	
**Water intake**								<0.001
As before	1,043 (38)	94 (35.9)	544 (39.6)	116 (42.6)	185 (38.9)	27 (29.7)	77 (28.5)	
Lower	707 (25.8)	34 (13)	400 (29.1)	64 (23.5)	127 (26.7)	26 (28.6)	56 (20.7)	
Higher	995 (36.2)	134 (51.1)	430 (31.3)	92 (33.8)	164 (34.5)	38 (41.8)	137 (50.7)	
**Hygiene measures**								0.068
As before	466 (17)	31 (11.8)	239 (17.4)	47 (17.3)	94 (19.7)	18 (19.8)	37 (13.7)	
Higher	2,279 (83)	231 (88.2)	1,135 (82.6)	225 (82.7)	382 (80.3)	73 (80.2)	233 (86.3)	
**Perishable foods**								0.309
As before	1,033 (37.6)	108 (41.2)	509 (37)	102 (37.5)	183 (38.4)	30 (33)	101 (37.4)	
Lower	334 (12.2)	24 (9.2)	153 (11.1)	38 (14)	64 (13.4)	17 (18.7)	38 (14.1)	
Higher	1,378 (50.2)	130 (49.6)	712 (51.8)	132 (48.5)	229 (48.1)	44 (48.4)	131 (48.5)	
**Expenditure on food**								0.010
As Before	544 (19.8)	65 (24.8)	273 (19.9)	59 (21.7)	89 (18.7)	14 (15.4)	44 (16.3)	
Lower	251 (9.2)	11 (4.2)	148 (10.8)	28 (10.3)	38 (8)	7 (7.7)	19 (7)	
Higher	1,950 (71.0)	186 (71)	953 (69.4)	185 (68)	349 (73.3)	70 (76.9)	207 (76.7)	
**Difficult to find food**								<0.001
No	1,831 (66.7)	168 (64.1)	968 (70.5)	182 (66.9)	300 (63)	43 (47.3)	170 (63)	
Yes	914 (33.3)	94 (35.9)	406 (29.5)	90 (33.1)	176 (37)	48 (52.7)	100 (37)	
**Frequency of home-cooking**								0.001
Not before, but now	196 (7.1)	20 (7.63)	104 (7.57)	17 (6.25)	31 (6.51)	5 (5.49)	19 (7.04)	
Never	158 (5.8)	14 (5.34)	79 (5.75)	19 (6.99)	29 (6.09)	1 (1.10)	16 (5.93)	
As before	640 (23.3)	88 (33.6)	285 (20.7)	70 (25.7)	115 (24.2)	17 (18.7)	65 (24.1)	
Lower	124 (4.5)	26 (9.92)	39 (2.84)	20 (7.35)	19 (3.99)	9 (9.89)	11 (4.07)	
Higher	1,627 (59.3)	114 (43.5)	867 (63.1)	146 (53.7)	282 (59.2)	59 (64.8)	159 (58.9)	

a Differences between groups were evaluated by chi-squared-test.

b *Number of meals out of home before the quarantine*.

Table 4 shows dietary behaviors by culinary processes and regions during the confinement. There were no significant differences observed in the frequency of frying food by regions (*p*-value = 0.254), but for other culinary processes: boiling (*p*-value = 0.02), baking (*p*-value < 0.001), microwaving (*p*-value = 0.02), stewing (*p*-value = 0.02), and griddling (*p*-value = 0.03). Specifically, boiling (47.5%) and griddling (40%) were the most often applied processes (4–5 points on the 0–5 frequency scale). Lower-frequency levels (3–2–1 points) were also reported for boiling (47.8%), frying (72.3%), and baking, stewing, and griddling (between 51.3 and 53.1%). Microwaving and baking were the less used culinary processes; 75.1 and 34.8% of respondents, respectively, never used it.

**Table 4:** Dietary behaviors of the diet-COVID-19 survey respondents in Colombia by regions according to the culinary processes applied.

**Process**		**National**	**Atlántica**	**Bogotá**	**Central**	**Oriental**	**Orinoquía and Amazonas**	**Pacífica**	***p-*value^**b**^**
**applied^**a**^**		***N* = 2,745 (%)**	***N* = 262 (%)**	***N* = 1,374 (%)**	***N* = 272 (%)**	***N* = 476 (%)**	***N* = 91 (%)**	***N* = 270 (%)**	
Boiled	0	131 (4.8)	19 (7.3)	53 (3.9)	16 (5.9)	17 (3.6)	6 (6.6)	20 (7.4)	0.016
	1	267 (9.7)	31 (11.8)	123 (9)	31 (11.4)	44 (9.2)	8 (8.8)	30 (11.1)	
	2	524 (19.1)	54 (20.6)	264 (19.2)	47 (17.3)	94 (19.7)	13 (14.3)	52 (19.3)	
	3	522 (19)	48 (18.3)	245 (17.8)	54 (19.9)	84 (17.6)	28 (30.8)	63 (23.3)	
	4	471 (17.2)	45 (17.2)	244 (17.8)	52 (19.1)	80 (16.8)	12 (13.2)	38 (14.1)	
	5	830 (30.2)	65 (24.8)	445 (32.4)	72 (26.5)	157 (33)	24 (26.4)	67 (24.8)	
Fried	0	392 (14.3)	31 (11.8)	207 (15.1)	41 (15.1)	65 (13.7)	5 (5.5)	43 (15.9)	0.254
	1	838 (30.5)	71 (27.1)	410 (29.8)	95 (34.9)	151 (31.7)	27 (29.7)	84 (31.1)	
	2	621 (22.6)	63 (24)	303 (22.1)	69 (25.4)	105 (22.1)	24 (26.4)	57 (21.1)	
	3	528 (19.2)	57 (21.8)	268 (19.5)	43 (15.8)	95 (20)	17 (18.7)	48 (17.8)	
	4	242 (8.8)	25 (9.5)	127 (9.2)	15 (5.5)	42 (8.8)	9 (9.9)	24 (8.9)	
	5	124 (4.5)	15 (5.7)	59 (4.3)	9 (3.3)	18 (3.8)	9 (9.9)	14 (5.2)	
Baked	0	955 (34.8)	122 (46.6)	430 (31.3)	87 (32)	166 (34.9)	50 (54.9)	100 (37)	<0.001
	1	517 (18.8)	39 (14.9)	265 (19.3)	44 (16.2)	106 (22.3)	15 (16.5)	48 (17.8)	
	2	464 (16.9)	40 (15.3)	236 (17.2)	52 (19.1)	91 (19.1)	8 (8.8)	37 (13.7)	
	3	427 (15.6)	35 (13.4)	214 (15.6)	50 (18.4)	68 (14.3)	12 (13.2)	48 (17.8)	
	4	227 (8.3)	14 (5.3)	136 (9.9)	23 (8.5)	31 (6.5)	5 (5.5)	18 (6.7)	
	5	155 (5.6)	12 (4.6)	93 (6.8)	16 (5.9)	14 (2.9)	1 (1.1)	19 (7)	
Microwave	0	2,068 (75.3)	210 (80.2)	996 (72.5)	199 (73.2)	386 (81.1)	77 (84.6)	200 (74.1)	0.022
	1	303 (11)	25 (9.5)	160 (11.6)	28 (10.3)	47 (9.9)	8 (8.8)	35 (13)	
	2	168 (6.1)	13 (5.0)	101 (7.4)	19 (7)	17 (3.6)	3 (3.3)	15 (5.6)	
	3	109 (4.0)	8 (3.1)	55 (4.0)	18 (6.6)	14 (2.9)	2 (2.2)	12 (4.4)	
	4	51 (1.9)	3 (1.1)	31 (2.3)	2 (0.7)	7 (1.5)	1 (1.1)	7 (2.6)	
	5	46 (1.7)	3 (1.1)	31 (2.3)	6 (2.2)	5 (1.1)	0 (0)	1 (0.4)	
Stew	0	694 (25.3)	86 (32.8)	313 (22.8)	83 (30.5)	121 (25.4)	31 (34.1)	60 (22.2)	0.017
	1	426 (15.5)	38 (14.5)	197 (14.3)	49 (18)	69 (14.5)	14 (15.4)	59 (21.9)	
	2	554 (20.2)	48 (18.3)	291 (21.2)	47 (17.3)	99 (20.8)	18 (19.8)	51 (18.9)	
	3	477 (17.4)	40 (15.3)	264 (19.2)	40 (14.7)	72 (15.1)	10 (11)	51 (18.9)	
	4	379 (13.8)	31 (11.8)	197 (14.3)	36 (13.2)	74 (15.5)	11 (12.1)	30 (11.1)	
	5	215 (7.8)	19 (7.3)	112 (8.2)	17 (6.3)	41 (8.6)	7 (7.7)	19 (7)	
Griddle	0	199 (7.2)	26 (9.9)	86 (6.3)	26 (9.6)	31 (6.5)	10 (11)	20 (7.4)	0.027
	1	364 (13.3)	34 (13)	164 (11.9)	41 (15.1)	73 (15.3)	15 (16.5)	37 (13.7)	
	2	569 (20.7)	49 (18.7)	292 (21.3)	49 (18)	107 (22.5)	24 (26.4)	48 (17.8)	
	3	519 (18.9)	50 (19.1)	266 (19.4)	38 (14)	93 (19.5)	14 (15.4)	58 (21.5)	
	4	565 (20.6)	45 (17.2)	301 (21.9)	53 (19.5)	103 (21.6)	15 (16.5)	48 (17.8)	
	5	529 (19.3)	58 (22.1)	265 (19.3)	65 (23.9)	69 (14.5)	13 (14.3)	59 (21.9)	

a Culinary processes applied: include a 5-point scale ranging from “never” (0) to “very often” (5).

b *Differences between groups were evaluated by chi-squared-test*.

Table 5 shows dietary behaviors concerning fried food consumption by regions. While fried food consumption frequency was found to be similar between the regions (Table 4), we observed that the change in the consumption of these foods before and during the confinement differed significantly between them. The largest increase in fried consumption during the confinement was found in Orinoquía and Amazonas (38.5%), and the lowest in Bogotá (18.5%), the differences in the change being statistically significant between the regions (*p-*value < 0.001). Half of the respondents consumed fried food one to three times/week. Over 6% of respondents from Orinoquía and Amazonas reported to eat fried food more than seven times/week compared with <2% of respondents from the other regions (*p-*value = 0.002). Also, stir-fry frequency was higher in this region (25% of respondents, more than five times/week) compared with the others (*p-*value < 0.001). The most consumed oil for frying was sunflower oil followed by canola and soybean oils. Also, oil type consumption differed significantly between the regions (*p-*value < 0.001). Oil reutilization was more uncommon in Bogotá (48.3% of respondents) but frequently applied in Orinoquía and Amazonas (85.7% of respondents) (*p-*value < 0.001).

**Table 5:** Fried food consumption before and during confinement among the diet-COVID-19 survey respondents in Colombia by regions.

	**National**	**Atlántica**	**Bogotá**	**Central**	**Oriental**	**Orinoquía and Amazonas**	**Pacífica**	***p*-value^**a**^**
	***N* = 2,745 (%)**	***N =* 262 (%)**	***N =* 1,374 (%)**	***N =* 272 (%)**	***N =* 476 (%)**	***N* = 91 (%)**	***N* = 270 (%)**	
**Fried food frequency**								<0.001
Never	341 (12.4)	27 (10.3)	187 (13.6)	35 (12.9)	44 (9.2)	9 (9.9)	39 (14.4)	
As before	1,354 (49.3)	117 (44.7)	682 (49.6)	134 (49.3)	253 (53.2)	38 (41.8)	130 (48.1)	
Lower	511 (18.6)	62 (23.7)	251 (18.3)	61 (22.4)	84 (17.6)	9 (9.9)	44 (16.3)	
Higher	539 (19.6)	56 (21.4)	254 (18.5)	42 (15.4)	95 (20)	35 (38.5)	57 (21.1)	
**Fried food intake**								0.002
Never or <1 times/week	900 (32.8)	81 (30.9)	472 (34.4)	96 (35.3)	134 (28.8)	20 (19.9)	94 (34.8)	
1–3 times/week	1,429 (52.1)	140 (53.4)	720 (52.4)	136 (50)	261 (54.8)	44 (48.4)	128 (47.4)	
4–6 times/week	371 (13.5)	40 (15.3)	164 (11.9)	36 (13.2)	67 (14.1)	21 (23.1)	43 (15.9)	
≥7 times/week	45 (1.6)	1 (0.4)	18 (1.3)	4 (1.5)	11 (2.3)	6 (6.6)	5 (1.9)	
**Stir-fry frequency**								<0.001
<3 times/week	1,008 (36,7)	118 (45.0)	465 (33.8)	129 (47.4)	149 (31.3)	26 (28.6)	121 (44.8)	
3–4 times/week	1,114 (40.6)	111 (42.4)	577 (42.0)	81 (29.8)	188 (39.5)	42 (46.2)	115 (42.6)	
≥5 times/week	623 (22,7)	33 (12.6)	332 (24.2)	62 (22.8)	139 (29.2)	23 (25.3)	34 (12.6)	
**Oil type**^**b**^								<0.001
Sunflower oil	1,144 (41.7)	103 (39.3)	604 (44)	104 (38.2)	204 (42.9)	24 (26.4)	105 (38.9)	
Canola oil	315 (11.5)	17 (6.5)	170 (12.4)	37 (13.6)	62 (13)	3 (3.3)	26 (9.6)	
Corn oil	59 (2.1)	10 (3.8)	25 (1.8)	4 (1.5)	11 (2.3)	1 (1.1)	8 (3)	
Olive oil	327 (11.9)	18 (6.9)	193 (14)	33 (12.1)	50 (10.5)	6 (6.6)	27 (10)	
Palm oil	90 (3.3)	19 (7.3)	24 (1.7)	13 (4.8)	18 (3.8)	5 (5.5)	11 (4.1)	
Soy bean oil	279 (10.2)	38 (14.5)	93 (6.8)	35 (12.9)	47 (9.9)	26 (28.6)	40 (14.8)	
Mixed oils	246 (9)	33 (12.6)	109 (7.9)	19 (7)	46 (9.7)	16 (17.6)	23 (8.5)	
Other	196 (7.1)	1 (0.4)	56 (4.1)	7 (2.6)	15 (3.2)	3 (3.3)	7 (2.6)	
UK	89 (3.2)	23 (8.8)	100 (7.3)	20 (7.4)	23 (4.8)	7 (7.7)	23 (8.5)	
**Oil reutilization**								<0.001
Never	1,114 (40.6)	66 (25.2)	663 (48.3)	92 (33.8)	194 (40.8)	13 (14.3)	86 (31.9)	
2 times	1,082 (39.4)	133 (50.8)	498 (36.2)	107 (39.3)	196 (41.2)	41 (45.1)	107 (39.6)	
≥3 times	388 (14.1)	47 (17.9)	141 (10.3)	53 (19.5)	68 (14.3)	29 (31.9)	50 (18.5)	
UK	161 (5.9)	16 (6.1)	72 (5.2)	20 (7.4)	18 (3.8)	8 (8.8)	27 (10)	

a Differences between groups were evaluated by chi-squared-test.

b Refers to oil type used for frying and other culinary processes.

*UK, unknown*.

### Dietary Patterns at National and Region Level

**Figure 1:**
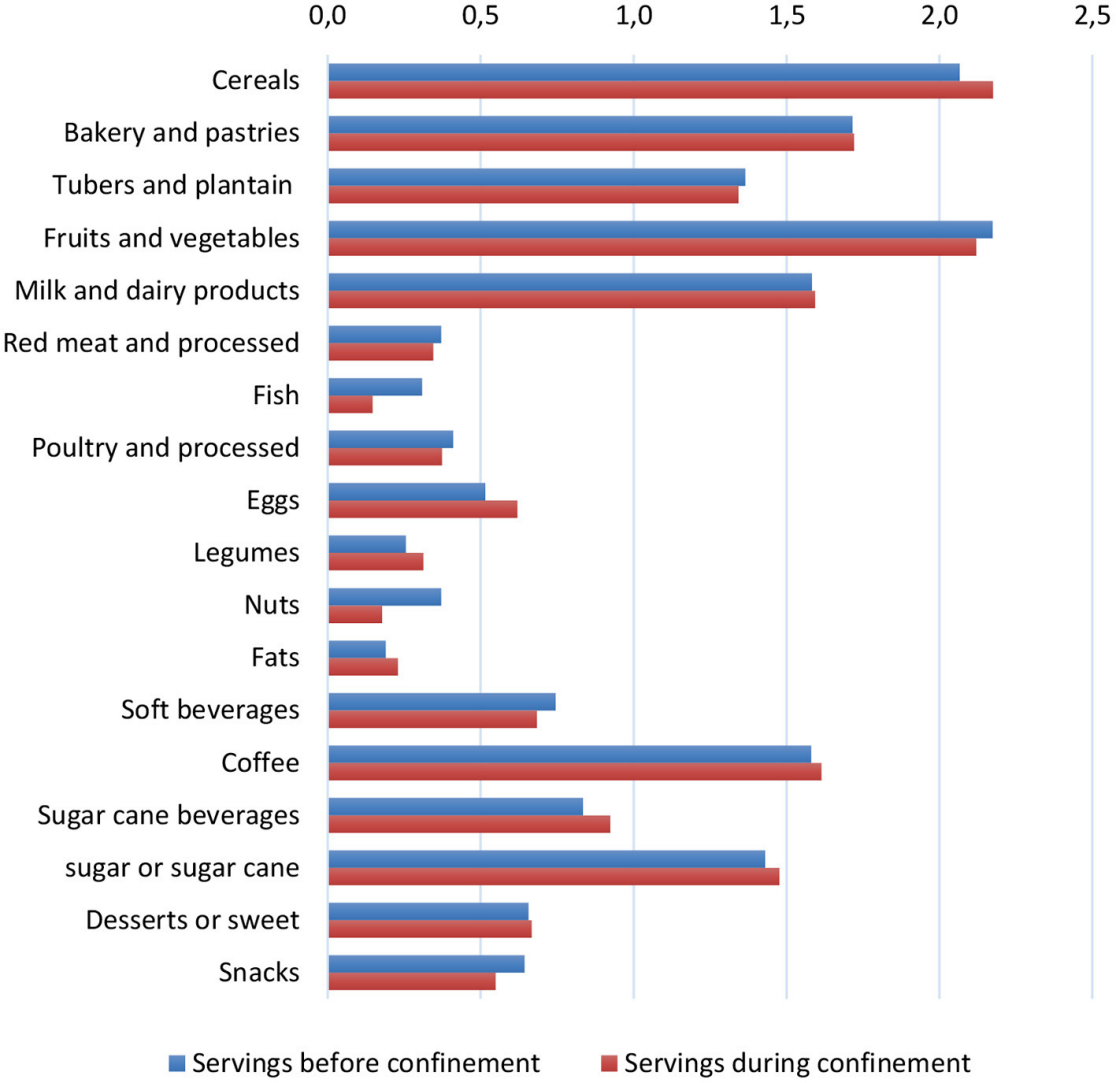
Bar plots showing consumption of main food groups in servings/day among the diet-COVID-19 survey respondents in Colombia, before and during the confinement. Information on dietary consumption of main food groups during the confinement was gathered by the respondents in servings per days. Consumption before the confinement was estimated considering information on whether consumption was alike, higher, or lower during confinement than before (similar servings, one serving less and one serving more, respectively).

**Figure 2:**
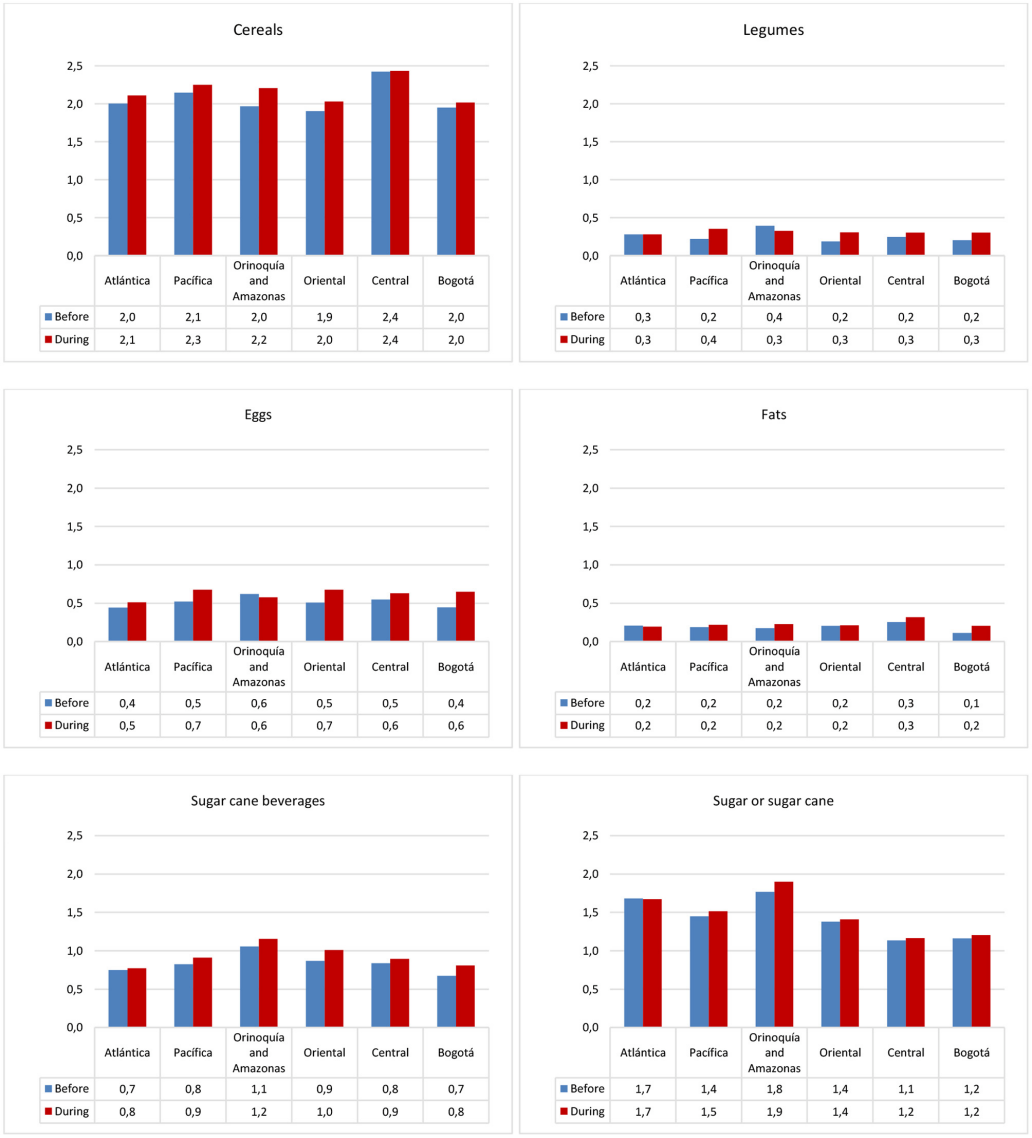
Bar plots showing consumption of food groups in servings/day among the diet-COVID-19 survey respondents in Colombia by regions, before and during the confinement. Only food groups that increased more importantly during the confinement are shown.

Figure 1 shows the average daily serving consumption of 18 food groups before and during the confinement at the country level. Comparing the two situations, the consumption pattern seemed to change more importantly with regard to cereals, legumes, eggs, fats, coffee, sugar or sugar cane, and their beverages; their intake increased notably during the confinement. This change was seen in all regions although to a varying extent (Supplementary Figure 1). Particularly with regard to cereals, during the confinement, their consumption was reported to be as usual by 60–70% of the respondents, while over 20% of them reported an increase in their consumption (Supplementary Figure 2). As shown in Figure 2, this increased consumption resulted in a higher amount of servings/day during the confinement in all regions. On the other hand, intake of other food groups, such as fish and nuts, decreased in all regions during the confinement; that of fruits and vegetables seemed to decline in every region, except in Bogotá and Central regions (Supplementary Figure 2). Nonetheless, during the confinement, significant differences by regions in the dietary intake of all foods were observed, except for snacks (Supplementary Table 1).

**Figure 3:**
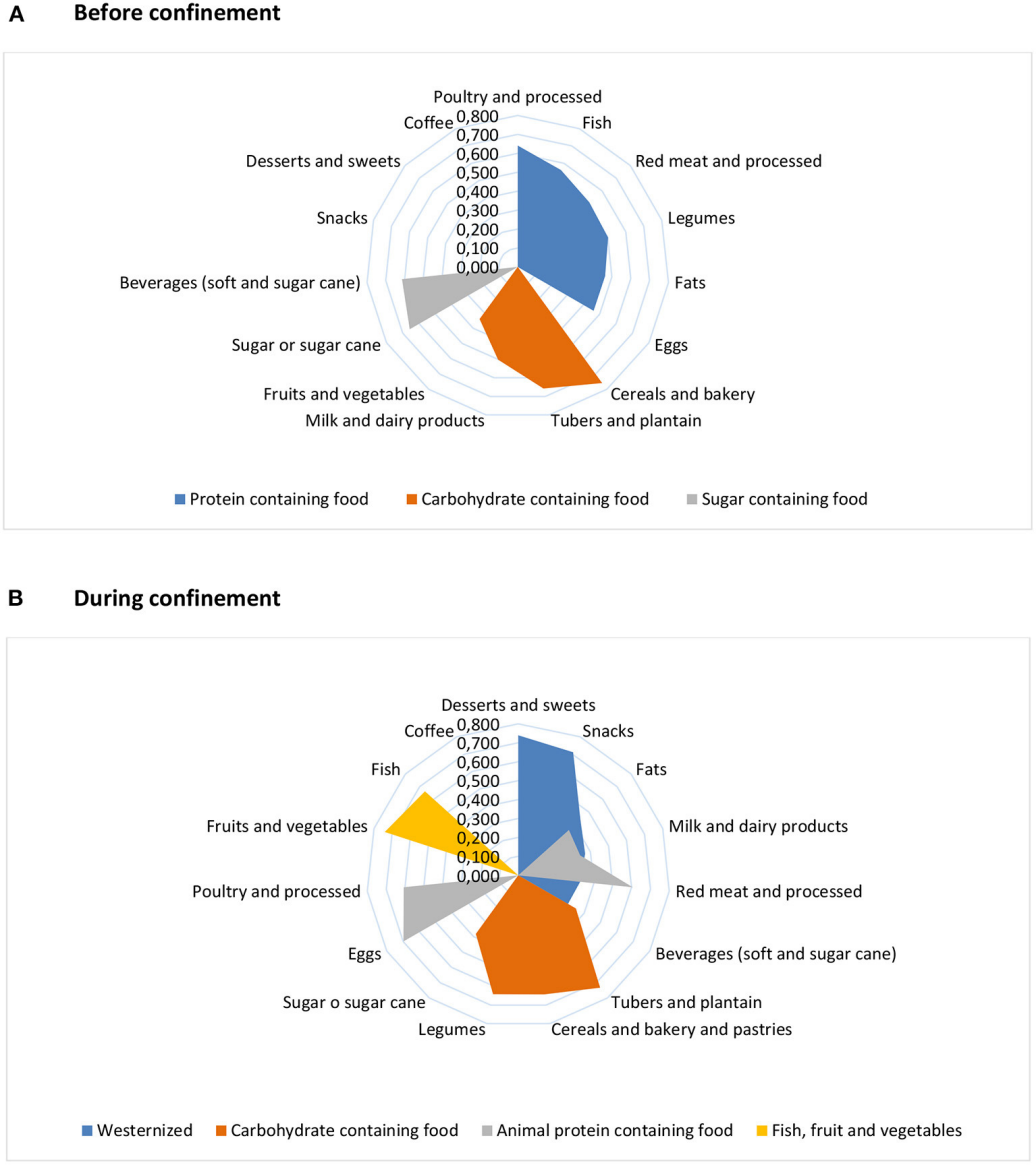
Radial charts showing dietary clusters derived from principal component analysis among the diet-COVID-19 survey respondents in Colombia, before **(A)** and during **(B)** the confinement. Factor loadings (>0.3) are presented along the *x*-axis. Information on dietary consumption of main food groups during the confinement was gathered by the respondents in servings per days or weeks. Consumption before the confinement was estimated considering information on whether consumption was alike, higher, or lower during confinement than before (similar servings, one serving less, and one serving more, respectively). Dietary intake in grams/day was estimated by applying standard portion sizes of each food.

Figure 3 shows food intake in the form of dietary clusters derived from PCA, similarly before and during the confinement. We observed three main clusters (i.e., DPs) before confinement: a protein-rich and carbohydrate-rich dietary cluster characterized by high intakes of food sources of these nutrients, and a sugary dietary cluster characterized by high positive loadings (>0.3) on soft beverages, sugar, and cane, but negative loadings (>-0.3) on fruits and vegetables. During the confinement, the protein and carbohydrate dietary clusters remained to a certain extent, albeit with some modifications, mostly in the carbohydrate cluster. The latter incorporated sugared foods such as sugar and sugar cane, and beverages, whereas other food components were depleted (fruits and vegetables, and milk and dairy products). In addition, two new clusters emerged: a westernized-diet-like cluster scoring positively for soft beverages, sugar and fats, snacks, milk and dairy products, red meat and processed, and desserts, and another cluster characterized by positive loadings on fruits, vegetables, and fish. Factor loadings of foods in each cluster and explained variance of the dietary clusters are shown in Supplementary Table 2. The explained variance was highest for the protein-rich cluster before the confinement and for the westernized dietary cluster during the confinement, both followed by the carbohydrate-rich dietary cluster. Dietary clusters were relatively stable during the survey (Supplementary Figure 3); they remained similar with regard to the protein-rich and carbohydrate-rich dietary clusters in the early confinement and later. Remarkably, the fruits and vegetable and fish dietary cluster appeared from the fourth week of confinement.

By regions (Supplementary Figure 4), we observed that all showed the carbohydrate-rich dietary cluster before the confinement, but with some differences. For instance, the cluster was similar among most regions, but snacks were part of this dietary cluster in Atlántica, and Orinoquía and Amazonas showed a mixed cluster with cereals, bakery, soft beverages, and fish. This dietary cluster became more diversified during the confinement in some regions: tubers and plantains with cereals and bakery constituted a separate component in Atlántica and Bogotá regions, while cereals and bakery clustered with some protein foods in Orinoquía and Amazonas, Central, and Pacifica regions. The fruits and vegetable, and fish dietary cluster emerged in Bogotá, Central, and Oriental regions, as well as in Atlántica and Pacífica, albeit with some variations. More details on the changes in factor loadings from pre-to-post-confinement are shown in Supplementary Figure 5.

Adherence scores to the nationally derived dietary clusters also varied by regions (Supplementary Table 3). No consistent differences in adherence by sex, age, or other variables observed (data not shown). Before the confinement, the Orinoquía and Amazonas region showed a higher adherence to the protein and sugary dietary clusters compared with the other regions (*p*-value = 0.03 and *p-*value < 0.001, respectively). During the confinement, however, adherence to the carbohydrate-rich dietary clusters was highest for Orinoquía and Amazonas and Pacifica compared with the others (*p-*value < 0.001).

**Figure 4:**
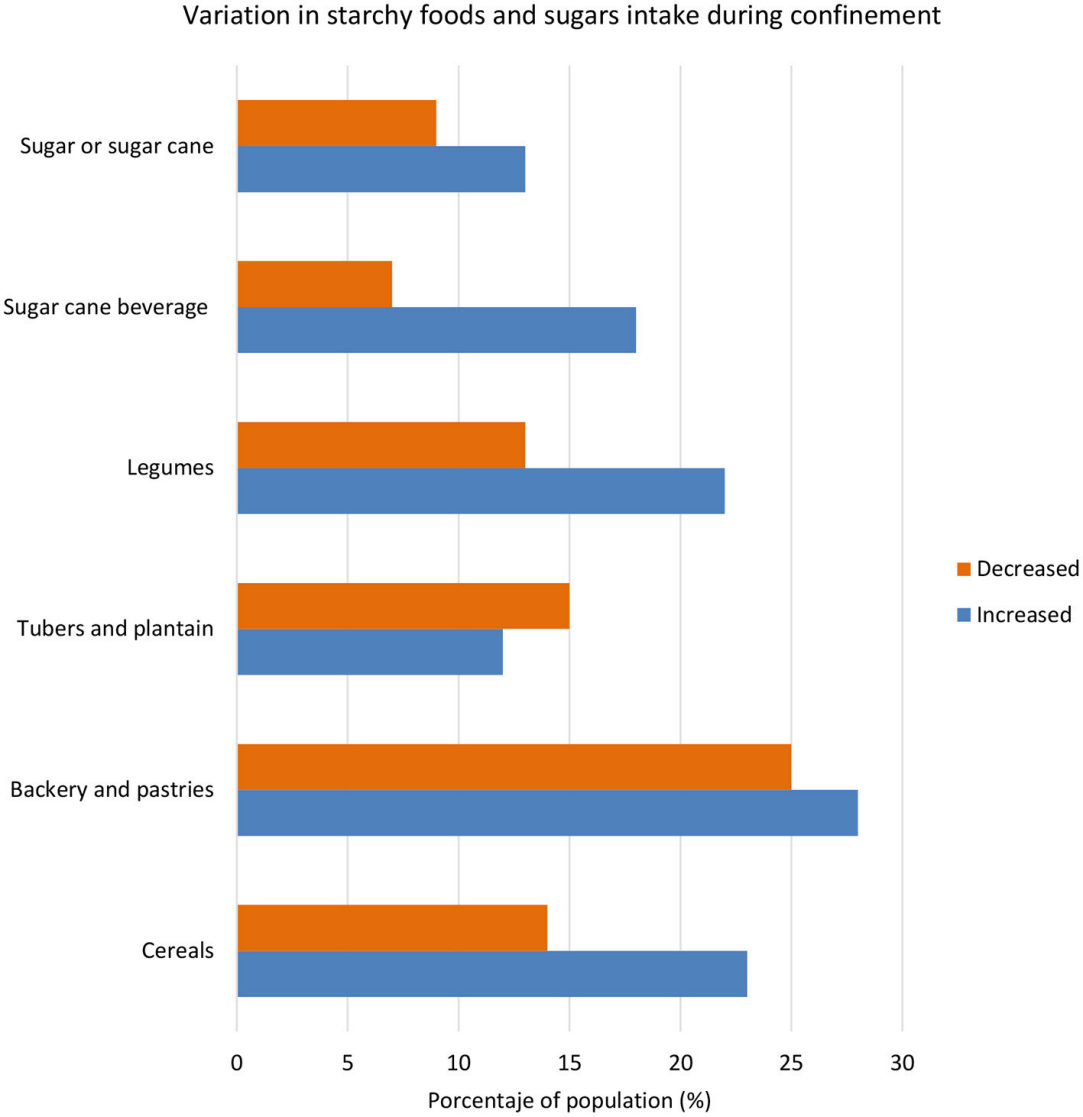
Variation in consumption of starchy foods and sugars during confinement when compared with previous intake among the diet-COVID-19 survey respondents (% of respondonts) in Colombia.

Given the importance of the carbohydrate-rich dietary cluster, we analyzed further the variation of consumption of starchy foods and sugar from pre-to-post-confinement (Figure 4). Overall, a higher proportion of participants reported to have increased the consumption of these foods during confinement, except that of tubers and plantains. The largest increase was observed for sugar cane beverages (from around 7 to 18%). Despite this increase, almost half of the respondents were non-consumers of these beverages during the confinement, and 40% did not consume any sugar or sugar cane. In contrast, over half of the respondents consumed one serving per day of tubers and plantains, and 20–30% of them consumed one serving per day of all other starchy foods and sugar (Supplementary Table 1).

## Discussion

The present study, aimed at evaluating the impact of the COVID-19 confinement on the dietary behaviors of a convenience sample of the Colombian population at the national and regional levels, shows that there were important regional differences with regard to these domains. Specifically, cooking frequency and snacking between meals increased during the confinement and was found to differ substantially between the regions. Conversely, frequency of frying remained stable but with considerable differences across regions. Dietary habits in the form of food intake or as DPs, prior to and during the confinement, also differed between the different regions. Importantly, the COVID-19 confinement led to an overall higher consumption of cereals, legumes, eggs, fats, and sugar and sugar cane and its beverages and lower consumptions of fish and nuts, as reflected by a different shape of DPs when compared with those present before the confinement. These changes underscore the fact that a transition toward the consumption of several unhealthy foods took place during the confinement.

Although significant differences were found between the regions with regard to the consumption of foods during the confinement, we observed a general trend toward an increased intake of cereals, eggs, fats, sugars, and sugar cane. The majority of the study population (>90%) reported that, during the confinement, they regularly consumed cereals, bakery and pastries, and tubers and plantains, these food groups being the base of their diet. An important proportion of this population (>60%) also consumed poultry and red and processed meat, eggs, legumes, fruits and vegetables, milk and dairy products, fish, coffee, and fats, and approximately one-half of them consumed sugar, cane and cane beverages, sweetened beverages, snacks, and alcoholic beverages. Together, this consumption profile is slightly different to those reported by the country's National Nutritional Surveys ENSIN (22) and the Latin American Study of Nutrition and Health ELANS (23). For instance, over 70% of the study participants consumed fish, compared with 21% of the Colombian population in the ELANS study (23). It is also noteworthy that there were fewer consumers (between 36 and 61%) of food groups with added sugars (desserts and sweets, sugar or cane sugar, sugar beverages, soft beverages) than that of basic foods. In particular, only 61% referred to consume sugar or cane sugar and 52% consumed traditional sugar cane beverages, known as “water with panela.” In this study population, the prevalence of snack eaters was also low (40%). This may, in part, be due to the fact that the studied population included mostly young aged women of high educational level, possibly more likely to make healthier food choices.

Overall, but with certain regional differences, the confinement also implied an increase in the practice of home-cooking (59.3%) and a greater purchase of perishable foods (50%), both being possibly associated with a maintenance or decrease (33.8%) in the consumption of fast foods and carbonated beverages, sodas, and soft drinks with respect to the pre-COVID-19 confinement period. However, about half of the participant reported to have increased their snacking frequency, to eat more and to practice less physical activity during the confinement. Conversely, a high proportion of participants were unaware of weight gain (40%), and only 22.3% reported to have gained weight. In Colombia, the increasing prevalence of overweight and obesity is of great concern due to its association with non-communicable chronic diseases, which contribute significantly to the morbidity and mortality of the country's adult population (8, 24, 25). In fact, the prevalence of overweight and obesity in Colombian adults aged over 18 years is 34.6 and 16.5%, respectively, these rates being higher in women. In the surveyed population, these proportions were 30.5 and 9.1%, respectively; only 18.5% reported to have modified their diet due to health issues.

With regard to culinary processes applied during the confinement, boiling was the most commonly reported one, followed by griddling. However, frying is a culinary technique widely used in the Colombian gastronomy. It ranks second after boiling of traditional preparations or meals and the main oils used are sunflower, soy, and palm and mixtures of palm with other oils. The frequency per day reported by the ENSIN survey was 0.5 serving/day (8). By contrast, in our study, fried foods were consumed between one and three times/week by more than half of the population, and <1 time or not consumed by a third of the population, suggesting that our study population adopted healthier cooking practices. Also, sunflower oil was more frequently used by the participants. These findings may be related to behavioral characteristics of our study population.

We have identified three main DPs before the confinement by considering the reported consumption and its change due to the confinement: the protein-rich, the carbohydrate-rich and the sugary DPs. In a cross-sectional study of 37,667 persons aged 5–64 years within the ENSIN survey from 2010, there were also three DPs identified: traditional/starch pattern, fruit-vegetable/dairy patterns, and snack pattern (26). In a subsequent study on these patterns on the ENSIN 2015 data (27), it was found that adherence to these three patterns was maintained. However, the adherence to these patterns was lower in adults (aged 27–64 years) than in children, adolescents, and young adults, suggesting that the patterns were driven by the younger population. While our study population is not directly comparable as it comprised adults only, mostly below 35 years, with high levels of education and women, and despite we did not coincide in timing, the DPs resembled to some extent those reported by the ENSIN survey. With regard to the timing, it is also important to highlight that food import policies over time are likely to have played a role in the population's eating behavior. The following are major differences between our study and the ENSIN survey to be aware of: (1) the carbohydrate-rich pattern included fruits and vegetables, milk and dairy products, cereals, and tubers and plantains, this pattern being similar to the fruit-vegetable/dairy and the traditional/starch patterns in ENSIN. (2) We identified an additional DP of protein-rich foods, of which some foods (red and processed meat, and fats) were part of the snack pattern in ENSIN. This pattern reflects the higher consumption of meats, eggs, fish, and fats of our study population, inasmuch intakes of these foods are adopted by those of a greater attained educational level. (3) Sweetened beverages and sugars clustered in our study into the sugary pattern, whereas these foods were included in the snack pattern in ENSIN. Adherence to this snack pattern seemed to decrease between 2010 and 2015 (27), which may explain why this DP was not prevailing our study. Indeed, only 40% of our population consumed snacks during the confinement. It is also worth noting that the ENSIN study collected dietary data by means of a food frequency questionnaire of 30 food items. Similar to our study, our questionnaire inquired about the frequency of intake of 28 foods or food groups, typically consumed in Colombia. Besides, the ENSIN surveys also showed regional variations with regard to these DPs, especially with regard to the fruit-vegetable/dairy and the traditional/starch patterns (27). Both were found to be predominant in the Northwestern region and the Andean and South-Central regions, respectively. We also found differences in the patterns by regions, which were also apparent when examining adherence to the DPs in each region. For instance, adherence to the protein-rich and sugary DPs was highest in the Orinoquía and Amazonas region.

During the confinement, there was a change in the aforementioned DPs toward an unhealthier DP, i.e., a Westernized-like DP. This pattern was more prominent in the Central region. Interestingly, we identified the fish, fruits, and vegetable DP, which seemed to have a greater presence in the Bogotá region. Importantly, this DP emerged more importantly from the fourth week of confinement. At that time, the Easter period took place and this is known to change the nation's eating habits since fish consumption typically increases to accommodate dietary requests of the Catholic Holy Week. This was probably more likely in regions subjected to receiving fresh food products during the confinement. On the other hand, the protein-rich and carbohydrate-rich food pattern seemed to be relatively constant, except that the latter became more sugared by incorporating sugar or sugar cane and beverages while losing components such as fruits and vegetables. Thus, this study shows that a Westernized DP predominated during the confinement in Colombia. This pattern is common in more developed countries, but in Colombia a transition from the traditional food pattern to a more Westernized pattern is already underway according to the ENSIN surveys, although with regional differences (26, 27). This nutritional transition goes along with demographic changes in the country, with both driving dietary behavior changes (23). The increasing prevalence of the overweight and obesity, indeed, has been attributed to the adoption of poorer dietary habits of the population (28, 29). Furthermore, there are important variations by regions with regard to social inequalities and poverty, with the Pacifica and Southeast regions being the most affected, and thus more prone to experience such food transitions. Our study reveals that the Westernized pattern and the carbohydrate-rich foods pattern were the strongest ones during the confinement. Overall, the regions Orinoquía and Amazonas (Southeast region), Central, and Pacifica showed the highest adherence to these patterns, whereas Bogotá showed the lowest. Hence, this result is consistent with the above. Moreover, these two DPs featured not only the consumption of unhealthy foods but also of foods most often fried or cooked with oils of all kinds. Sweetened beverages, cane and cane beverages were also major components of these DPs. Altogether, these foods, have been associated with detrimental health effects and risk of developing chronic diseases in numerous studies (30–35); e.g., with type 2 diabetes, obesity, metabolic syndrome, and cancer. Likewise, the Westernized DP and some types of carbohydrate-rich DPs (36), mostly those rich in glycemic foods, have been associated with an increased risk of these diseases too (37, 38). Such DPs are also likely to affect the nutritional and immunological state of the population owing to their low content of essential nutrients (39–41), thereby possibly increasing the risk infectious diseases and cancer. This negative effect might be mediated by various mechanisms through the human microbiome (42). Indeed, a healthy balanced diet is related to gut microbe symbiosis, which in turn plays a decisive role on the immune response against the virus, whereas unhealthy DPs lead to microbial dysbiosis underlying the inactivation of the host immune response (42, 43). Also, several studies have claimed that dietary micronutrients with anti-inflammatory and immune-modulating potential are key to prevent COVID-19 disease (44–47).

There are several limitations to note. Firstly, as stated before, the study population was not representative of the Colombian population; extrapolation of the findings to the general population is therefore limited. In fact, the majority of the survey's respondents were young adults and women of high educational level. Secondly, the sample size was relatively small and there were fewer respondents in some regions, such as in Orinoquía and Amazonas, which might imply that numbers were limited to extract significant results. Also, there were differences in the baseline characteristics of the study sample by regions. However, similar DPs were observed on a homogenous subset of the study population (360 subjects of the same age, BMI and educational level) (data not shown).

Regarding strengths, the questionnaire was completed by all participants; thus, information bias is unlikely. Also, a large number of subjects from all the regions of the country participated in this study, making it possible to establish a nation-wide study and to make comparisons at the region level. Information on dietary behaviors during the confinement, and changes on the basis of prior habits, was collected in the form of an extensive questionnaire. At such a level of detail, we were able to analyze dietary behaviors related to cooking habits and others and to derive food consumption patterns before and during the confinement. Thus, this is the first study evaluating dietary behaviors related to culinary processes in Colombia, alongside changes in DPs during the COVID-19 confinement.

In conclusion, our study reveals that the COVID-19 confinement had a great impact on the dietary behaviors of a large adult population from Colombia. The fact that this population seemed to adopt less healthy dietary habits shows the need of evaluating this dietary transition in the near term and in the general population. This knowledge is essential for implementing appropriate public health nutrition interventions, aimed at promoting the consumption of healthy foods, of high nutritional value, together with varied and safety cooking practices, in the Colombian population during the COVID-19 pandemic and in a post-COVID stage.

## Data Availability Statement

The original contributions generated in the study are included in the article/Supplementary Material, further inquiries can be directed to the corresponding author.

## Ethics Statement

The studies involving human participants were reviewed and approved by The Human Research Ethics Committee of the University of Granada (1526/CEIH/2020) and is part of the COVIDiet-Int study (ClinicalTrials.gov Number NCT 04449731). Written informed consent for participation was not required for this study in accordance with the national legislation and the institutional requirements.

## Author Contributions

CR-P, MR-L, EM-M, VV, EG-H, RA, and BG-V formalized the theoretical framework and methodology. SP-C and EM-M contributed to data analysis. SP-C, EM-M, and BG-V wrote the original draft. BG-V supervised the whole work. All authors contributed to the review and editing of the final manuscript and have read and agreed the published version of the manuscript.

## Conflict of Interest

The authors declare that the research was conducted in the absence of any commercial or financial relationships that could be construed as a potential conflict of interest.
