# Supplementary material for: Exploring Dietary Behavior Changes Due to the COVID-19 Confinement in Colombia: A National and Regional Survey Study
Source: Front Nutr. 2021 Apr 12;8:644800. doi: 10.3389/fnut.2021.644800 (PMC8072011; doi:10.3389/fnut.2021.644800)
Supplement: Supplementary file 1 [file Data_Sheet_1.docx]

Supplementary Material

# Supplementary Tables

**Supplementary Table 1.** Consumption of main food groups during confinement in servings per days or week among the diet-COVID-19 survey respondents in Colombia by regions

|  | **National** | **Atlántica** | | **Bogotá** | **Central** | **Oriental** | **Orinoquía and Amazonas** | **Pacífica** | **p-value^1^** |
| --- | --- | --- | --- | --- | --- | --- | --- | --- | --- |
|  | N=2,745 (%) | N=262 (%) | N=1,374 (%) | | N=272 (%) | N=476 (%) | N=91 (%) | N=270 (%) |  |
| **Cereals servings/d** |  |  |  | |  |  |  |  | <0.001 |
| 0 | 46 (1.68) | 5 (1.91) | 28 (2.04) | | 2 (0.74) | 4 (0.84) | 1 (1.10) | 6 (2.22) |  |
| 1 | 819 (29.8) | 74 (28.2) | 426 (31.0) | | 71 (26.1) | 166 (34.9) | 27 (29.7) | 55 (20.4) |  |
| 2 | 1,076 (39.2) | 110 (42.0) | 556 (40.5) | | 91 (33.5) | 169 (35.5) | 35 (38.5) | 115 (42.6) |  |
| 3 | 511 (18.6) | 40 (15.3) | 250 (18.2) | | 50 (18.4) | 98 (20.6) | 13 (14.3) | 60 (22.2) |  |
| 4 | 206 (7.50) | 26 (9.92) | 86 (6.26) | | 31 (11.4) | 26 (5.46) | 10 (11.0) | 27 (10.0) |  |
| 5 | 87 (3.17) | 7 (2.67) | 28 (2.04) | | 27 (9.93) | 13 (2.73) | 5 (5.49) | 7 (2.59) |  |
| **Bakery and pastries servings/d** |  |  |  | |  |  |  |  | 0.001 |
| 0 | 250 (9.11) | 32 (12.2) | 110 (8.01) | | 15 (5.51) | 49 (10.3) | 7 (7.69) | 37 (13.7) |  |
| 1 | 991 (36.1) | 115 (43.9) | 483 (35.2) | | 91 (33.5) | 167 (35.1) | 38 (41.8) | 97 (35.9) |  |
| 2 | 906 (33.0) | 75 (28.6) | 443 (32.2) | | 112 (41.2) | 159 (33.4) | 25 (27.5) | 92 (34.1) |  |
| 3 | 399 (14.5) | 31 (11.8) | 222 (16.2) | | 35 (12.9) | 71 (14.9) | 12 (13.2) | 28 (10.4) |  |
| 4 | 140 (5.10) | 6 (2.29) | 80 (5.82) | | 12 (4.41) | 24 (5.04) | 7 (7.69) | 11 (4.07) |  |
| 5 | 59 (2.15) | 3 (1.15) | 36 (2.62) | | 7 (2.57) | 6 (1.26) | 2 (2.20) | 5 (1.85) |  |
| **Tubers and plantains servings/d** |  |  |  | |  |  |  |  | <0.001 |
| 0 | 235 (8.56) | 13 (4.96) | 142 (10.3) | | 16 (5.88) | 32 (6.72) | 7 (7.69) | 25 (9.26) |  |
| 1 | 1678 (61.1) | 166 (63.4) | 847 (61.6) | | 167 (61.4) | 293 (61.6) | 47 (51.6) | 158 (58.5) |  |
| 2 | 674 (24.6) | 65 (24.8) | 322 (23.4) | | 70 (25.7) | 123 (25.8) | 27 (29.7) | 67 (24.8) |  |
| 3 | 120 (4.37) | 13 (4.96) | 46 (3.35) | | 14 (5.15) | 25 (5.25) | 7 (7.69) | 15 (5.56) |  |
| 4 | 38 (1.38) | 5 (1.91) | 17 (1.24) | | 5 (1.84) | 3 (0.63) | 3 (3.30) | 5 (1.85) |  |
|  |  |  |  |  |  |  |  |  |  |
| **Fruits and vegetables servings/d** |  |  |  | |  |  |  |  | 0.026 |
| 0 | 131 (4.77) | 22 (8.40) | 44 (3.20) | | 13 (4.78) | 27 (5.67) | 14 (15.4) | 11 (4.07) |  |
| 1 | 716 (26.1) | 82 (31.3) | 343 (25.0) | | 67 (24.6) | 116 (24.4) | 29 (31.9) | 79 (29.3) |  |
| 2 | 833 (30.3) | 77 (29.4) | 419 (30.5) | | 88 (32.4) | 142 (29.8) | 23 (25.3) | 84 (31.1) |  |
| 3 | 671 (24.4) | 53 (20.2) | 351 (25.5) | | 52 (19.1) | 139 (29.2) | 14 (15.4) | 62 (23.0) |  |
| 4 | 256 (9.33) | 18 (6.87) | 136 (9.90) | | 35 (12.9) | 39 (8.19) | 8 (8.79) | 20 (7.41) |  |
| 5 | 138 (5.03) | 10 (3.82) | 81 (5.90) | | 17 (6.25) | 13 (2.73) | 3 (3.30) | 14 (5.19) |  |
| **Milk and dairy products servings/d** |  |  |  | |  |  |  |  | 0.015 |
| 0 | 272 (9.91) | 24 (9.16) | 132 (9.61) | | 29 (10.7) | 41 (8.61) | 18 (19.8) | 28 (10.4) |  |
| 1 | 1085 (39.5) | 121 (46.2) | 513 (37.3) | | 97 (35.7) | 214 (45.0) | 37 (40.7) | 103 (38.1) |  |
| 2 | 902 (32.9) | 82 (31.3) | 463 (33.7) | | 89 (32.7) | 156 (32.8) | 21 (23.1) | 91 (33.7) |  |
| 3 | 357 (13.0) | 27 (10.3) | 197 (14.3) | | 40 (14.7) | 48 (10.1) | 12 (13.2) | 33 (12.2) |  |
| 4 | 97 (3.53) | 3 (1.15) | 54 (3.93) | | 12 (4.41) | 15 (3.15) | 3 (3.30) | 10 (3.70) |  |
| 5 | 32 (1.17) | 5 (1.91) | 15 (1.09) | | 5 (1.84) | 2 (0.42) | 0 (0.00) | 5 (1.85) |  |
| **Red meat and processed servings/wk** |  |  |  | |  |  |  |  | 0.008 |
| 0 | 293 (10.7) | 23 (8.78) | 157 (11.4) | | 33 (12.1) | 35 (7.35) | 8 (8.79) | 37 (13.7) |  |
| 1 | 556 (20.3) | 51 (19.5) | 286 (20.8) | | 51 (18.8) | 95 (20.0) | 21 (23.1) | 52 (19.3) |  |
| 2 | 690 (25.1) | 76 (29.0) | 334 (24.3) | | 58 (21.3) | 131 (27.5) | 19 (20.9) | 72 (26.7) |  |
| 3 | 572 (20.8) | 63 (24.0) | 282 (20.5) | | 46 (16.9) | 115 (24.2) | 15 (16.5) | 51 (18.9) |  |
| 4 | 293 (10.7) | 22 (8.40) | 142 (10.3) | | 34 (12.5) | 59 (12.4) | 11 (12.1) | 25 (9.26) |  |
| 5 | 341 (12.4) | 27 (10.3) | 173 (12.6) | | 50 (18.4) | 41 (8.61) | 17 (18.7) | 33 (12.2) |  |
| **Fish servings/wk** |  |  |  | |  |  |  |  | 0.035 |
| 0 | 783 (28.5) | 59 (22.5) | 360 (26.2) | | 91 (33.5) | 144 (30.3) | 33 (36.3) | 96 (35.6) |  |
| 1 | 1290 (47.0) | 139 (53.1) | 640 (46.6) | | 124 (45.6) | 236 (49.6) | 34 (37.4) | 117 (43.3) |  |
| 2 | 464 (16.9) | 46 (17.6) | 255 (18.6) | | 38 (14.0) | 67 (14.1) | 14 (15.4) | 44 (16.3) |  |
| 3 | 151 (5.50) | 14 (5.34) | 85 (6.19) | | 15 (5.51) | 22 (4.62) | 6 (6.59) | 9 (3.33) |  |
| 4 | 38 (1.38) | 2 (0.76) | 24 (1.75) | | 3 (1.10) | 5 (1.05) | 2 (2.20) | 2 (0.74) |  |
| 5 | 19 (0.69) | 2 (0.76) | 10 (0.73) | | 1 (0.37) | 2 (0.42) | 2 (2.20) | 2 (0.74) |  |
| **Poultry and processed servings/wk** |  |  |  | |  |  |  |  | <0.001 |
| 0 | 123 (4.48) | 12 (4.58) | 58 (4.22) | | 20 (7.35) | 12 (2.52) | 7 (7.69) | 14 (5.19) |  |
| 1 | 356 (13.0) | 48 (18.3) | 152 (11.1) | | 59 (21.7) | 51 (10.7) | 10 (11.0) | 36 (13.3) |  |
| 2 | 752 (27.4) | 75 (28.6) | 387 (28.2) | | 77 (28.3) | 132 (27.7) | 26 (28.6) | 55 (20.4) |  |
| 3 | 836 (30.5) | 81 (30.9) | 421 (30.6) | | 70 (25.7) | 144 (30.3) | 28 (30.8) | 92 (34.1) |  |
| 4 | 386 (14.1) | 25 (9.54) | 200 (14.6) | | 27 (9.93) | 87 (18.3) | 11 (12.1) | 36 (13.3) |  |
| 5 | 292 (10.6) | 21 (8.02) | 156 (11.4) | | 19 (6.99) | 50 (10.5) | 9 (9.89) | 37 (13.7) |  |
| **Eggs servings/wk** |  |  |  | |  |  |  |  | <0.001 |
| 0 | 66 (2.40) | 11 (4.20) | 28 (2.04) | | 13 (4.78) | 5 (1.05) | 2 (2.20) | 7 (2.59) |  |
| 1.5 | 449 (16.4) | 74 (28.2) | 207 (15.1) | | 48 (17.6) | 61 (12.8) | 24 (26.4) | 35 (13.0) |  |
| 3.5 | 834 (30.4) | 100 (38.2) | 428 (31.1) | | 73 (26.8) | 136 (28.6) | 29 (31.9) | 68 (25.2) |  |
| 5.5 | 720 (26.2) | 41 (15.6) | 361 (26.3) | | 62 (22.8) | 152 (31.9) | 15 (16.5) | 89 (33.0) |  |
| 7 | 676 (24.6) | 36 (13.7) | 350 (25.5) | | 76 (27.9) | 122 (25.6) | 21 (23.1) | 71 (26.3) |  |
| **Legumes servings/wk** |  |  |  | |  |  |  |  | 0.001 |
| 0 | 198 (7.21) | 19 (7.25) | 112 (8.15) | | 13 (4.78) | 38 (7.98) | 6 (6.59) | 10 (3.70) |  |
| 1 | 734 (26.7) | 69 (26.3) | 368 (26.8) | | 90 (33.1) | 130 (27.3) | 23 (25.3) | 54 (20.0) |  |
| 2 | 791 (28.8) | 100 (38.2) | 388 (28.2) | | 75 (27.6) | 126 (26.5) | 24 (26.4) | 78 (28.9) |  |
| 3 | 643 (23.4) | 58 (22.1) | 327 (23.8) | | 54 (19.9) | 111 (23.3) | 22 (24.2) | 71 (26.3) |  |
| 4 | 233 (8.49) | 7 (2.67) | 109 (7.93) | | 26 (9.56) | 46 (9.66) | 9 (9.89) | 36 (13.3) |  |
| 5 | 146 (5.32) | 9 (3.44) | 70 (5.09) | | 14 (5.15) | 25 (5.25) | 7 (7.69) | 21 (7.78) |  |
| **Nuts servings/wk** |  |  |  | |  |  |  |  | 0.004 |
| 0 | 1028 (37.4) | 120 (45.8) | 474 (34.5) | | 87 (32.0) | 205 (43.1) | 46 (50.5) | 96 (35.6) |  |
| 1 | 734 (26.7) | 69 (26.3) | 372 (27.1) | | 77 (28.3) | 118 (24.8) | 19 (20.9) | 79 (29.3) |  |
| 2 | 442 (16.1) | 38 (14.5) | 219 (15.9) | | 48 (17.6) | 74 (15.5) | 15 (16.5) | 48 (17.8) |  |
| 3 | 256 (9.33) | 14 (5.34) | 149 (10.8) | | 31 (11.4) | 40 (8.40) | 5 (5.49) | 17 (6.30) |  |
| 4 | 144 (5.25) | 9 (3.44) | 86 (6.26) | | 14 (5.15) | 15 (3.15) | 3 (3.30) | 17 (6.30) |  |
| 5 | 141 (5.14) | 12 (4.58) | 74 (5.39) | | 15 (5.51) | 24 (5.04) | 3 (3.30) | 13 (4.81) |  |
| **Fats servings/wk** |  |  |  | |  |  |  |  | <0.001 |
| 0 | 852 (31.0) | 77 (29.4) | 465 (33.8) | | 59 (21.7) | 142 (29.8) | 25 (27.5) | 84 (31.1) |  |
| 1 | 738 (26.9) | 83 (31.7) | 362 (26.3) | | 52 (19.1) | 143 (30.0) | 25 (27.5) | 73 (27.0) |  |
| 2 | 494 (18.0) | 54 (20.6) | 240 (17.5) | | 52 (19.1) | 82 (17.2) | 17 (18.7) | 49 (18.1) |  |
| 3 | 339 (12.3) | 28 (10.7) | 162 (11.8) | | 38 (14.0) | 64 (13.4) | 14 (15.4) | 33 (12.2) |  |
| 4 | 158 (5.76) | 14 (5.34) | 77 (5.60) | | 24 (8.82) | 24 (5.04) | 5 (5.49) | 14 (5.19) |  |
| 5 | 164 (5.97) | 6 (2.29) | 68 (4.95) | | 47 (17.3) | 21 (4.41) | 5 (5.49) | 17 (6.30) |  |
|  |  |  |  | |  |  |  |  |  |
| **Soft beverages servings/d** |  |  |  | |  |  |  |  | <0.001 |
| 0 | 1767 (64.4) | 142 (54.2) | 924 (67.2) | | 178 (65.4) | 305 (64.1) | 45 (49.5) | 173 (64.1) |  |
| 1 | 599 (21.8) | 70 (26.7) | 291 (21.2) | | 52 (19.1) | 111 (23.3) | 22 (24.2) | 53 (19.6) |  |
| 2 | 207 (7.54) | 26 (9.92) | 85 (6.19) | | 27 (9.93) | 30 (6.30) | 9 (9.89) | 30 (11.1) |  |
| 3 | 91 (3.32) | 13 (4.96) | 39 (2.84) | | 6 (2.21) | 23 (4.83) | 6 (6.59) | 4 (1.48) |  |
| 4 | 81 (2.95) | 11 (4.20) | 35 (2.55) | | 9 (3.31) | 7 (1.47) | 9 (9.89) | 10 (3.70) |  |
| **Coffee servings/d** |  |  |  | |  |  |  |  | <0.001 |
| 0 | 592 (21.6) | 72 (27.5) | 287 (20.9) | | 55 (20.2) | 94 (19.7) | 25 (27.5) | 59 (21.9) |  |
| 1 | 760 (27.7) | 99 (37.8) | 364 (26.5) | | 81 (29.8) | 114 (23.9) | 26 (28.6) | 76 (28.1) |  |
| 2 | 633 (23.1) | 55 (21.0) | 302 (22.0) | | 73 (26.8) | 111 (23.3) | 19 (20.9) | 73 (27.0) |  |
| 3 | 370 (13.5) | 17 (6.49) | 203 (14.8) | | 26 (9.56) | 82 (17.2) | 15 (16.5) | 27 (10.0) |  |
| 4 | 390 (14.2) | 19 (7.25) | 218 (15.9) | | 37 (13.6) | 75 (15.8) | 6 (6.59) | 35 (13.0) |  |
| **Sugar cane beverages servings/d** |  |  |  | |  |  |  |  | <0.001 |
| 0 | 1329 (48.4) | 126 (48.1) | 710 (51.7) | | 130 (47.8) | 203 (42.6) | 33 (36.3) | 127 (47.0) |  |
| 1 | 819 (29.8) | 92 (35.1) | 393 (28.6) | | 80 (29.4) | 156 (32.8) | 24 (26.4) | 74 (27.4) |  |
| 2 | 337 (12.3) | 27 (10.3) | 148 (10.8) | | 37 (13.6) | 58 (12.2) | 23 (25.3) | 44 (16.3) |  |
| 3 | 147 (5.36) | 12 (4.58) | 71 (5.17) | | 11 (4.04) | 28 (5.88) | 9 (9.89) | 16 (5.93) |  |
| 4 | 113 (4.12) | 5 (1.91) | 52 (3.78) | | 14 (5.15) | 31 (6.51) | 2 (2.20) | 9 (3.33) |  |
| **Sugar or sugar cane servings/d** |  |  |  | |  |  |  |  | <0.001 |
| 0 | 1078 (39.3) | 71 (27.1) | 596 (43.4) | | 119 (43.8) | 169 (35.5) | 23 (25.3) | 100 (37.0) |  |
| 1 | 589 (21.5) | 60 (22.9) | 296 (21.5) | | 59 (21.7) | 113 (23.7) | 19 (20.9) | 42 (15.6) |  |
| 2 | 535 (19.5) | 59 (22.5) | 249 (18.1) | | 53 (19.5) | 92 (19.3) | 18 (19.8) | 64 (23.7) |  |
| 3 | 290 (10.6) | 43 (16.4) | 122 (8.88) | | 20 (7.35) | 57 (12.0) | 16 (17.6) | 32 (11.9) |  |
| 4 | 133 (4.85) | 14 (5.34) | 61 (4.44) | | 13 (4.78) | 23 (4.83) | 5 (5.49) | 17 (6.30) |  |
| 5 | 120 (4.37) | 15 (5.73) | 50 (3.64) | | 8 (2.94) | 22 (4.62) | 10 (11.0) | 15 (5.56) |  |
| **Desserts and sweets servings/d** |  |  |  | |  |  |  |  | <0.001 |
| 0 | 1271 (46.3) | 146 (55.7) | 600 (43.7) | | 109 (40.1) | 233 (48.9) | 50 (54.9) | 133 (49.3) |  |
| 1 | 1169 (42.6) | 86 (32.8) | 626 (45.6) | | 139 (51.1) | 190 (39.9) | 22 (24.2) | 106 (39.3) |  |
| 2 | 228 (8.31) | 22 (8.40) | 107 (7.79) | | 17 (6.25) | 40 (8.40) | 14 (15.4) | 28 (10.4) |  |
| 3 | 77 (2.81) | 8 (3.05) | 41 (2.98) | | 7 (2.57) | 13 (2.73) | 5 (5.49) | 3 (1.11) |  |
| **Snacks servings/d** |  |  |  | |  |  |  |  | 0.087 |
| 0 | 1655 (60.3) | 158 (60.3) | 804 (58.5) | | 174 (64.0) | 304 (63.9) | 51 (56.0) | 164 (60.7) |  |
| 1 | 806 (29.4) | 71 (27.1) | 418 (30.4) | | 81 (29.8) | 133 (27.9) | 21 (23.1) | 82 (30.4) |  |
| 2 | 204 (7.43) | 26 (9.92) | 110 (8.01) | | 11 (4.04) | 25 (5.25) | 14 (15.4) | 18 (6.67) |  |
| 3 | 51 (1.86) | 4 (1.53) | 28 (2.04) | | 3 (1.10) | 9 (1.89) | 4 (4.40) | 3 (1.11) |  |
| 4 | 29 (1.06) | 3 (1.15) | 14 (1.02) | | 3 (1.10) | 5 (1.05) | 1 (1.10) | 3 (1.11) |  |
| ^1^ Differences between groups were evaluated by chi-squared test. Differences between groups were evaluate by Fisher’s exact test there were less than 5 observations in some categories. | | | | | | | | | |

**Supplementary Table 2.** Factor loadings for main foods denoting dietary clusters derived from principal component analysis among the diet-COVID-19 survey respondents in Colombia, before and during the confinement

|  | **Before** | | | **During** | | | |
| --- | --- | --- | --- | --- | --- | --- | --- |
|  | **Protein foods** | **Carbohydrate foods** | **Sugar foods** | **Westernized** | **Carbohydrate foods** | **Animal protein foods** | **Fish, fruits and vegetables** |
| Legumes | **0.5** | 0.22 | 0.23 | -0.14 | **0.64** | 0.04 | -0.04 |
| Cereals (cereals. bakery and pastries) | 0.11 | **0.76** | 0.01 | 0.24 | **0.64** | 0.16 | 0.1 |
| Beverages (soft beverages and sugar cane beverages) | 0.1 | 0.18 | **0.61** | **0.3** | **0.35** | -0.06 | -0.25 |
| Snacks (nuts and snacks) | 0.01 | 0.06 | 0.02 | **0.71** | 0.07 | -0.07 | 0.11 |
| Tubers and plantain | 0.14 | **0.66** | 0.25 | 0.13 | **0.73** | 0.04 | -0.01 |
| Fruits and vegetables | 0.06 | **0.34** | **-0.68** | 0.003 | 0.12 | 0.13 | **0.74** |
| Milk and dairy products | -0.001 | **0.5** | -0.21 | **0.37** | 0.12 | **0.34** | 0.24 |
| Red meat and processed | **0.51** | 0.09 | 0.09 | **0.33** | 0.02 | **0.61** | -0.27 |
| Fish | **0.56** | -0.13 | -0.14 | 0.14 | -0.08 | -0.01 | **0.66** |
| Poultry and processed | **0.64** | 0.06 | -0.06 | 0.02 | 0.01 | **0.61** | 0.17 |
| Eggs | **0.46** | 0.17 | -0.15 | -0.18 | 0.15 | **0.7** | 0.08 |
| Fats | **0.46** | 0.03 | 0.21 | **0.44** | 0.04 | **0.36** | -0.21 |
| Coffee | 0.01 | -0.06 | 0.08 | -0.07 | -0.1 | 0.14 | 0.14 |
| Sugar or sugar cane | 0.02 | 0.25 | **0.66** | 0.19 | **0.38** | 0.01 | **-0.43** |
| Desserts and sweets | 0.04 | 0.14 | 0.21 | **0.74** | 0.06 | 0.01 | 0.02 |
| **Explained variance** | **33%** | | | **45%** | | | |
| **Kaiser-Meyer-Olkin index** | **0.72** | | | **0.68** | | | |

Factor loadings higher than 3 are highlighted in bold.

**Supplementary Table 3:** Differences in scores of adherence to dietary patterns by regions, before and during the confinement

| **Region** | **Before** | | | **During** | | | |
| --- | --- | --- | --- | --- | --- | --- | --- |
|  | **Protein foods** | **Carbohydrate foods** | **Sugar foods** | **Westernized** | **Carbohydrate foods** | **Animal protein foods** | **Fish, fruits and vegetables** |
| Atlántica | -0.009 | -0.112 | 0.259 | -0.145 | -0.08 | -0.387 | -0.217 |
| Bogotá | -0.092 | -0.108 | -0.108 | -0.119 | -0.182 | 0.037 | 0.08 |
| Central | -0.078 | 0.167 | -0.041 | 0.029 | -0.072 | 0.083 | -0.227 |
| Oriental | -0.069 | -0.117 | -0.003 | -0.251 | -0.075 | 0.069 | -0.12 |
| Orinoquia y Amazonas | 0.246 | -0.065 | 0.347 | -0.038 | 0.188 | -0.284 | -0.43 |
| Pacífica | 0.005 | 0.05 | 0.072 | -0.262 | 0.186 | 0.146 | -0.223 |
| p-value^1^ | **0.003** | **0.001** | **<0.001** | **<0.001** | **<0.001** | **<0.001** | **<0.001** |

^1^ Within-region differences evaluated via Kruskal-Wallis Rank sum test with Welch´s heteroscedastic F test (one-way ANOVA for non-normal and heteroskedastic data). Pairwise-comparisons corrected via Bonferroni.

# Supplementary Figures

**Supplementary Figure 1.** Radial charts showing consumption of main food groups in servings/day among the diet-COVID-19 survey respondents in Colombia by regions. Before (A) and during (B) the confinement.

|  |  |
| --- | --- |
|  | 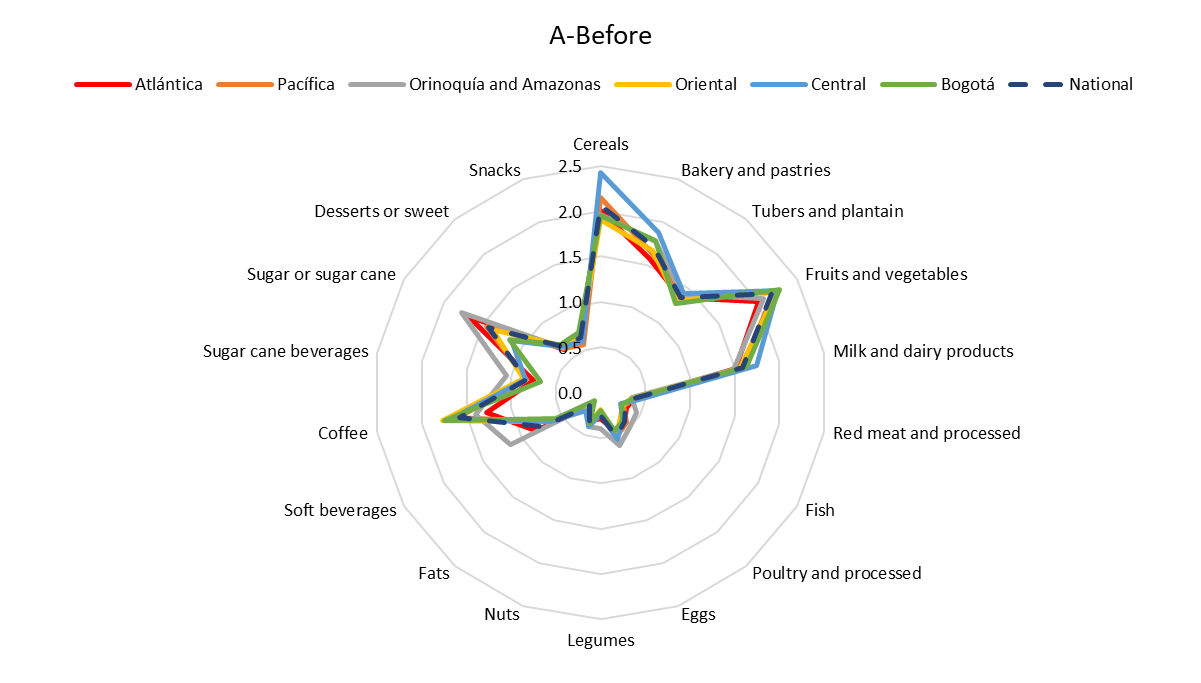 |
|  |  |
|  | 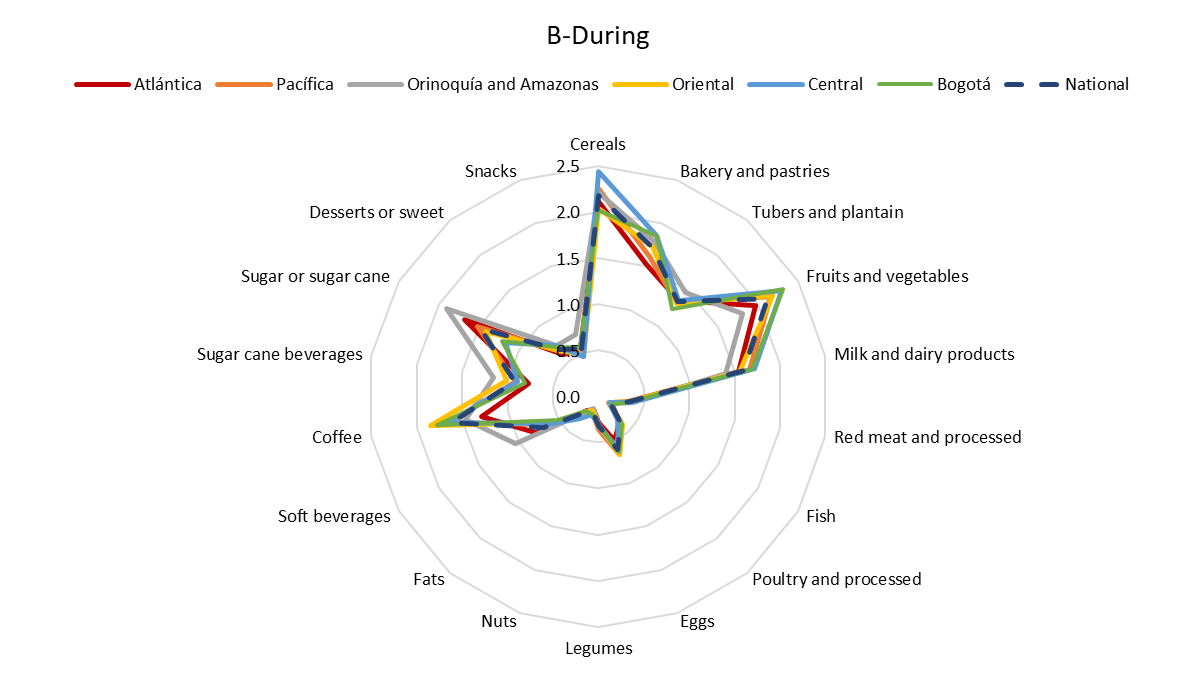 |

**Supplementary Figure 2.** Variation in consumption of main food groups during confinement when compared to previous dietary intake among the diet-COVID-19 survey respondents in Colombia by regions

| *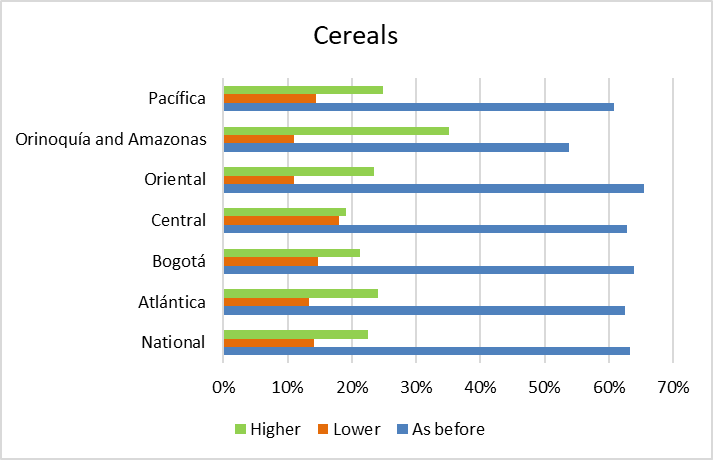* | *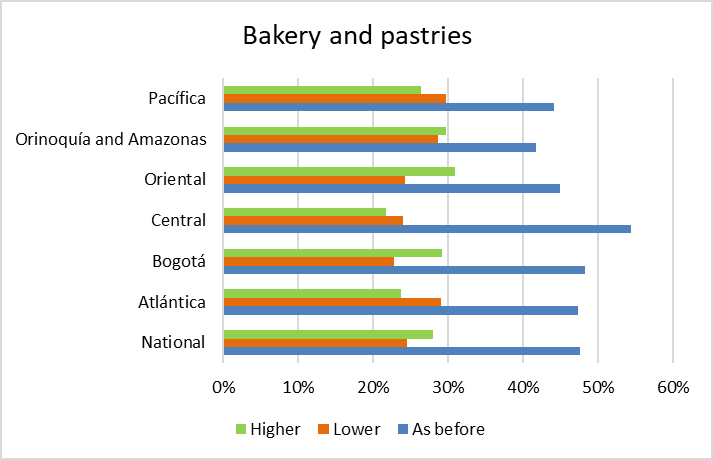* |
| --- | --- |
| 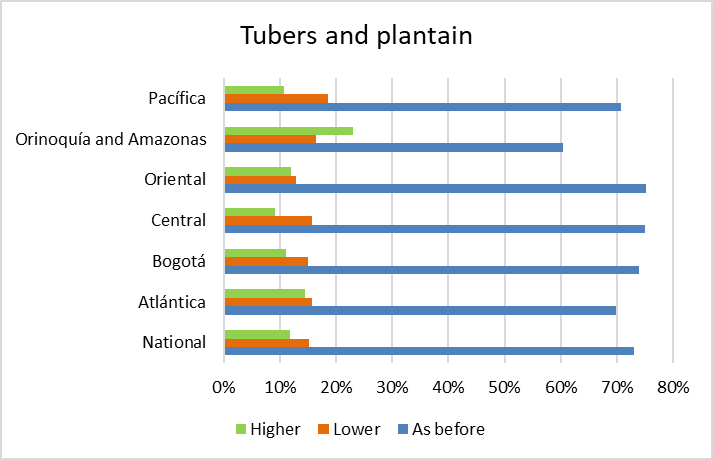 | 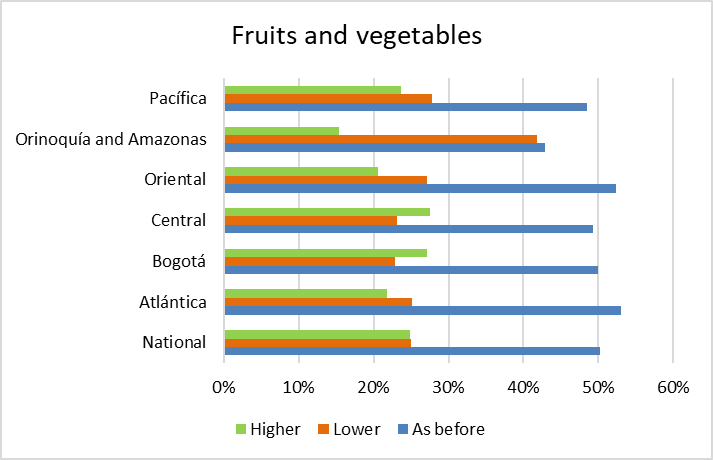 |
| 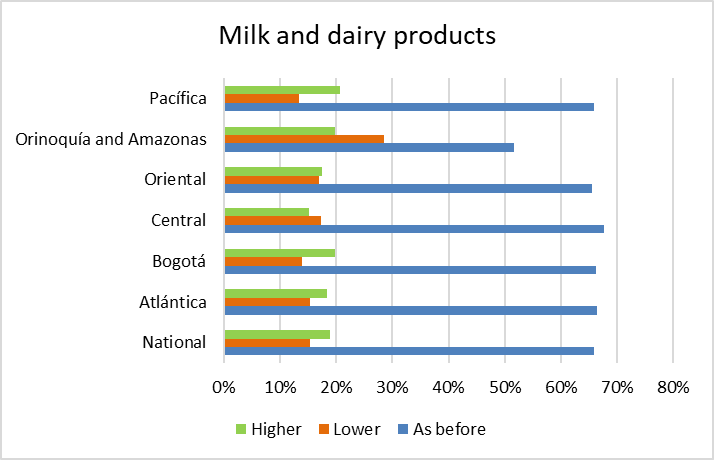 | 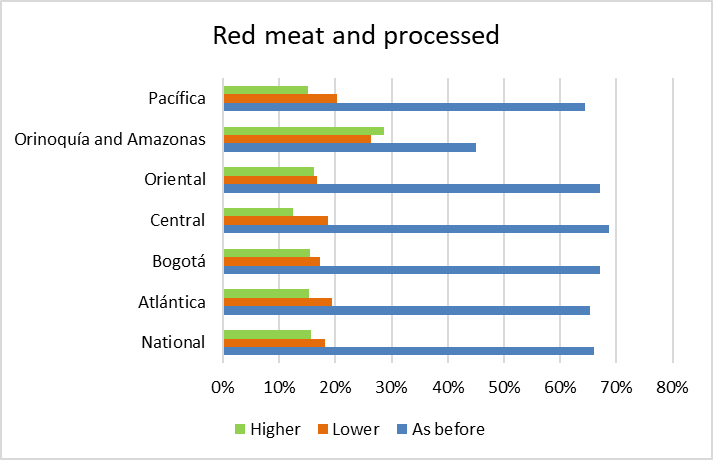 |
| 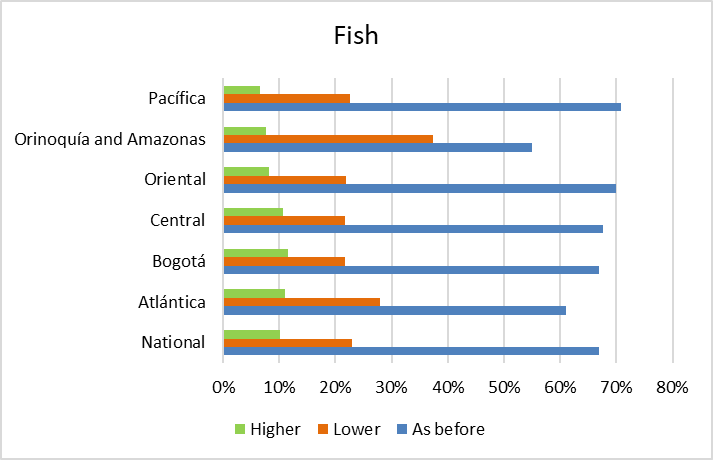 | 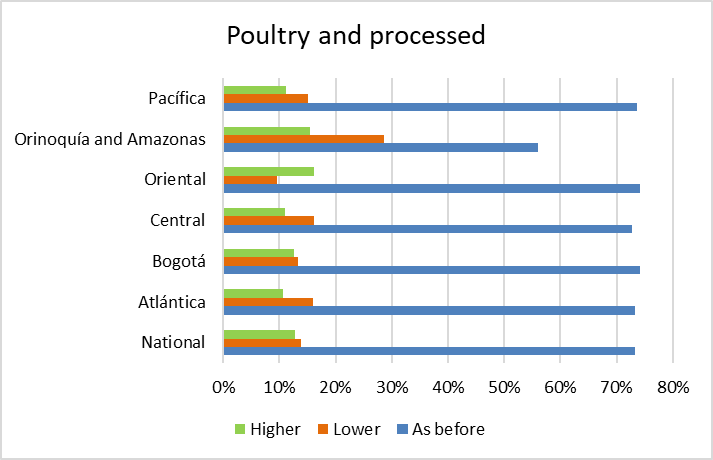 |
| 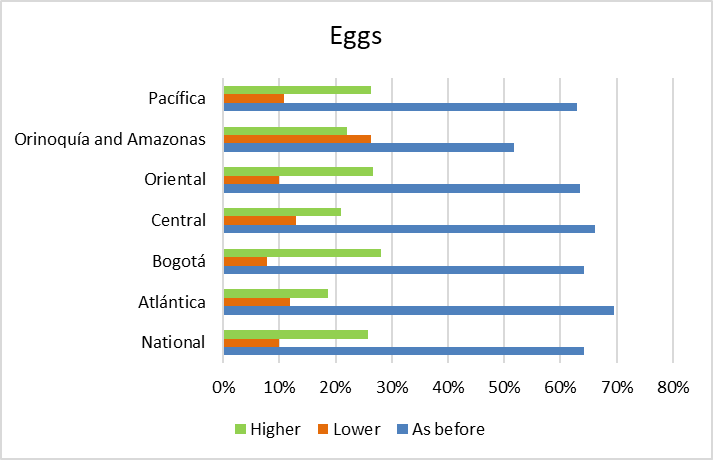 | 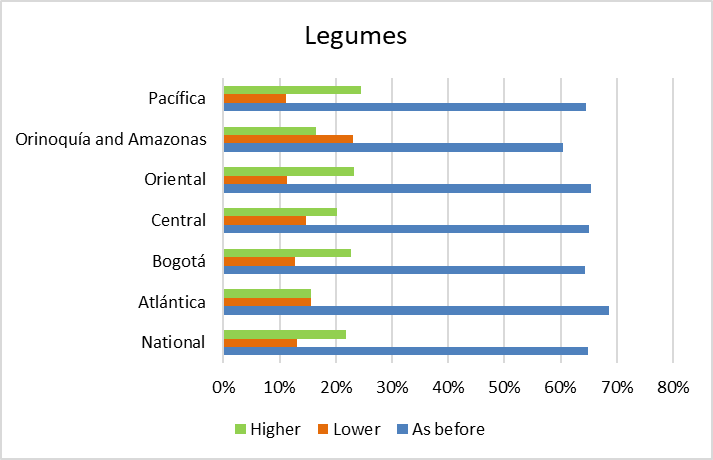 |
| 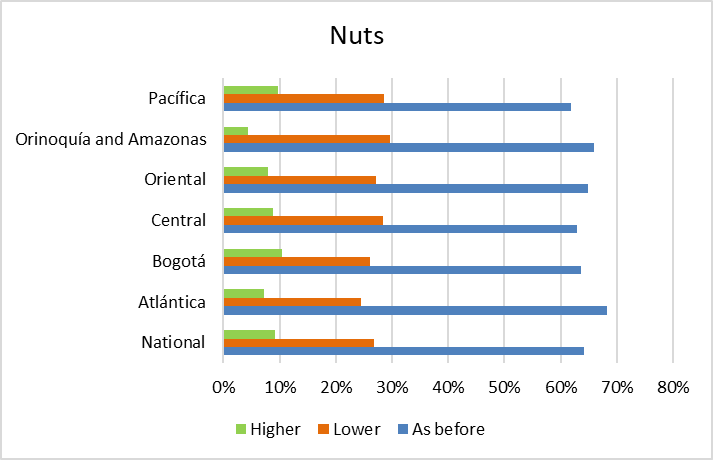 | 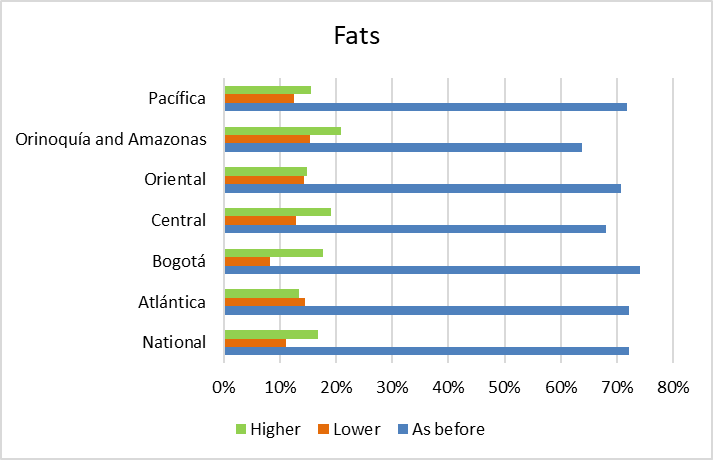 |
| 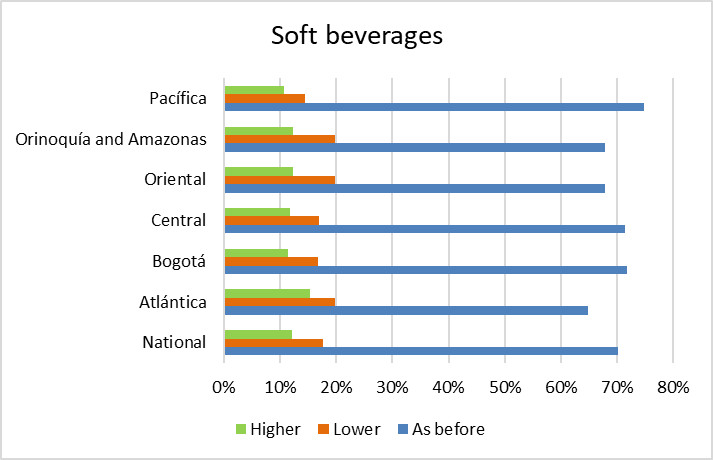 | 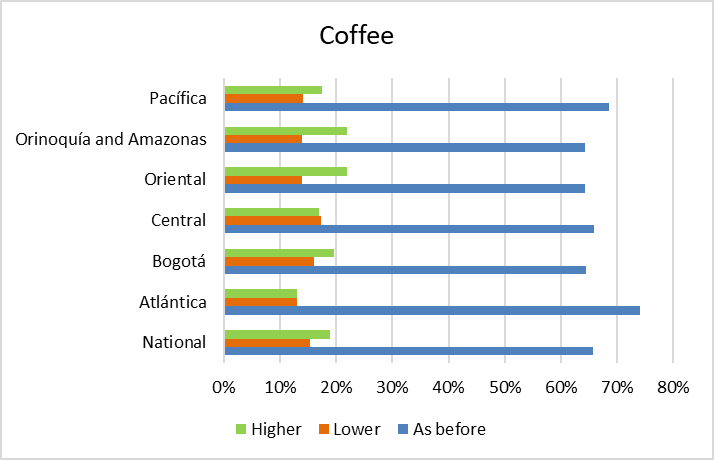 |
| 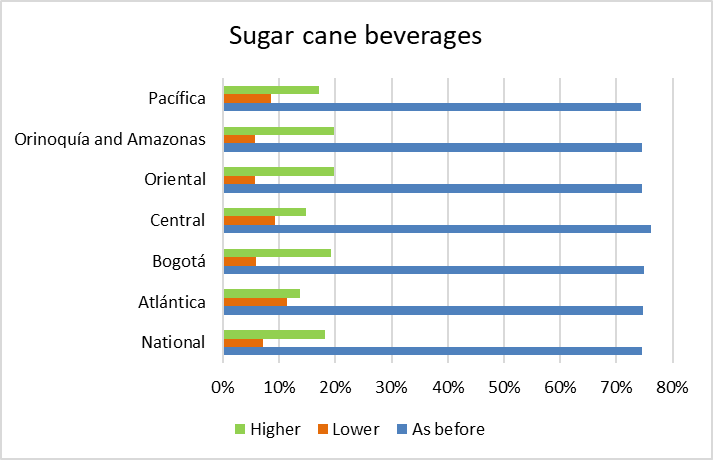 | 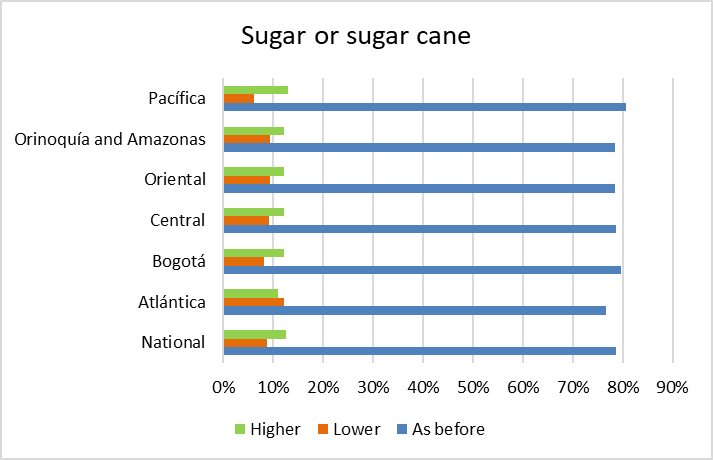 |
| 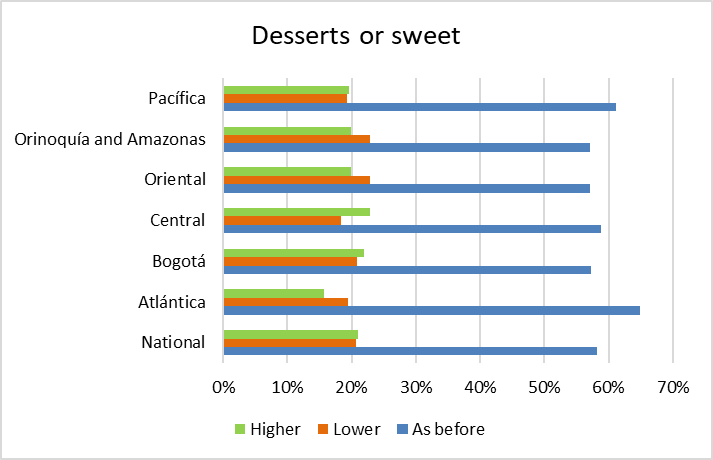 |  |

**Supplementary Figure 3.** Radial charts showing dietary clusters derived from principal component analysis among the diet-COVID-19 survey respondents in Columbia by weeks of confinement: from the second week (A) from the fourth week (B) of confinement.

|  | A-From the second week of confinement |
| --- | --- |
|  | 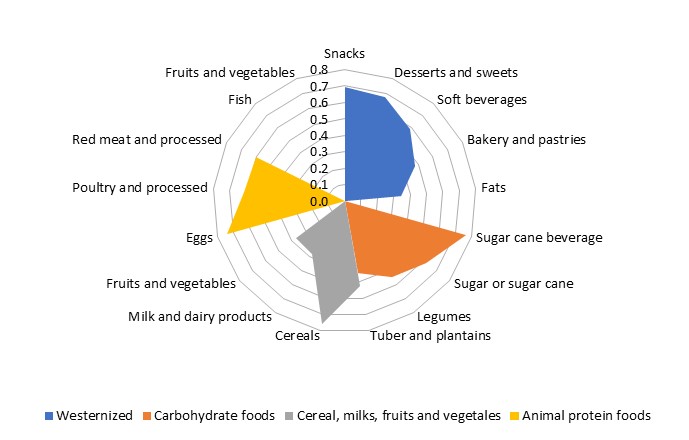 |
|  | B-From the fourth week of confinement |
|  | 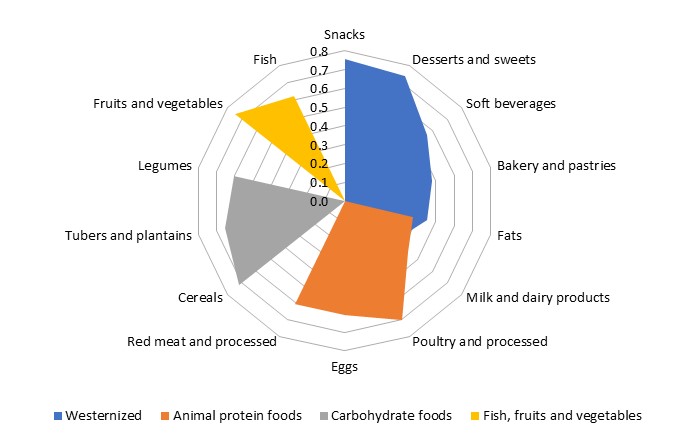 |

**Supplementary Figure 4.** Radial charts showing dietary clusters derived from principal component analysis among the diet-COVID-19 survey respondents in Columbia for every region (A – Atlántica; B – Bogota. C – Orinoquía and Amazonas. D – Central. E – Oriental. F – Pacifica), before and during the confinement

| A | Atlántica |  |
| --- | --- | --- |
|  | before | during |
|  | 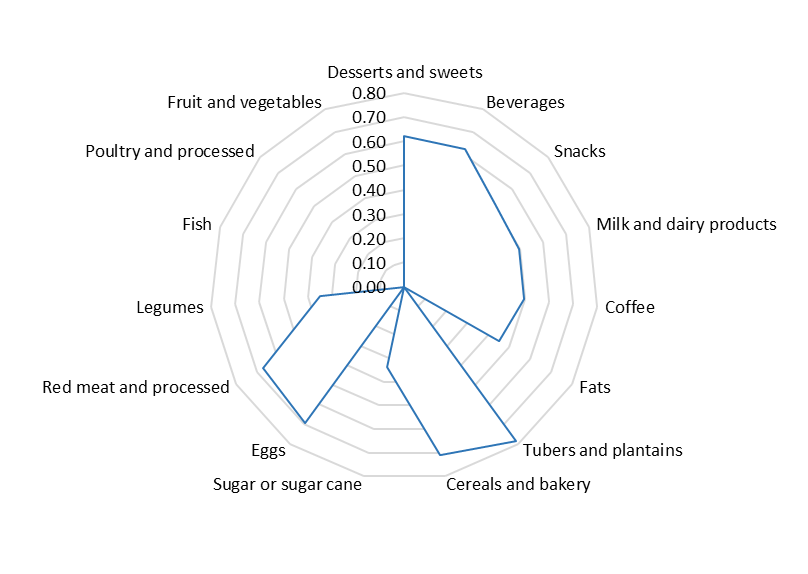 | 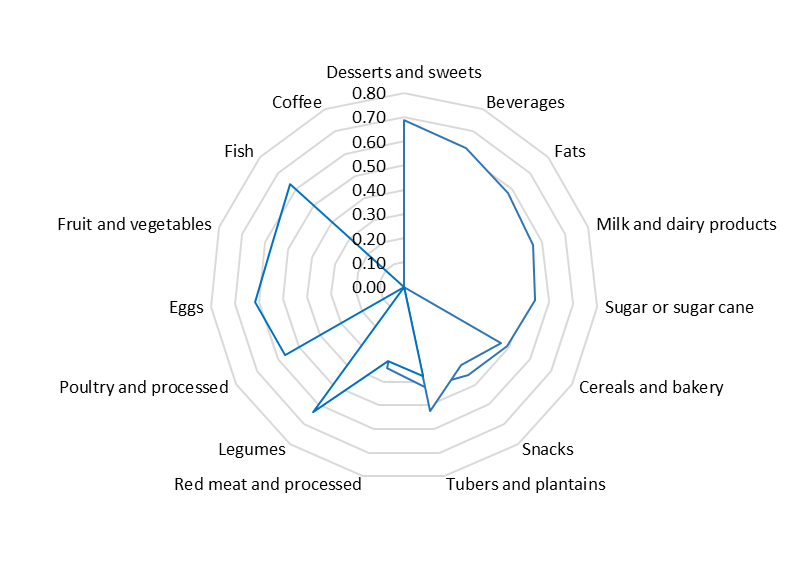 |
| B | Bogotá |  |
|  | before | during |
|  | 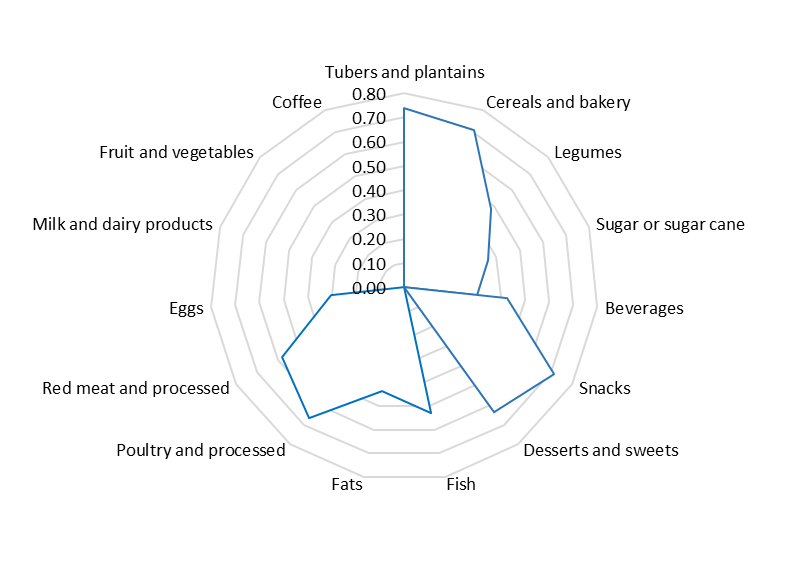 | 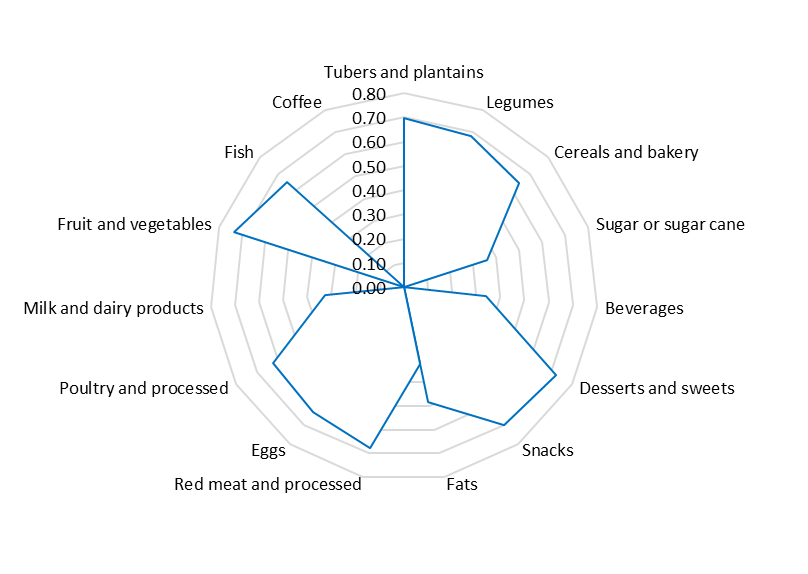 |
| C | Central |  |
|  | before | during |
|  | 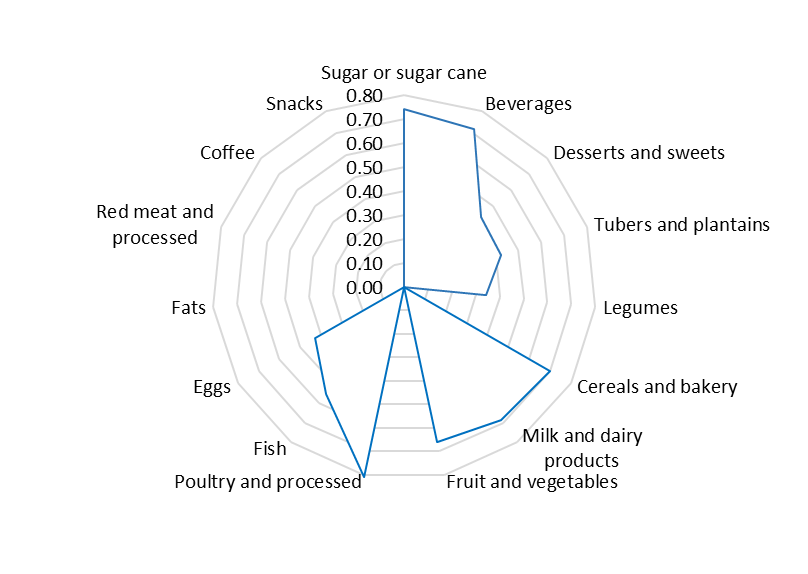 | 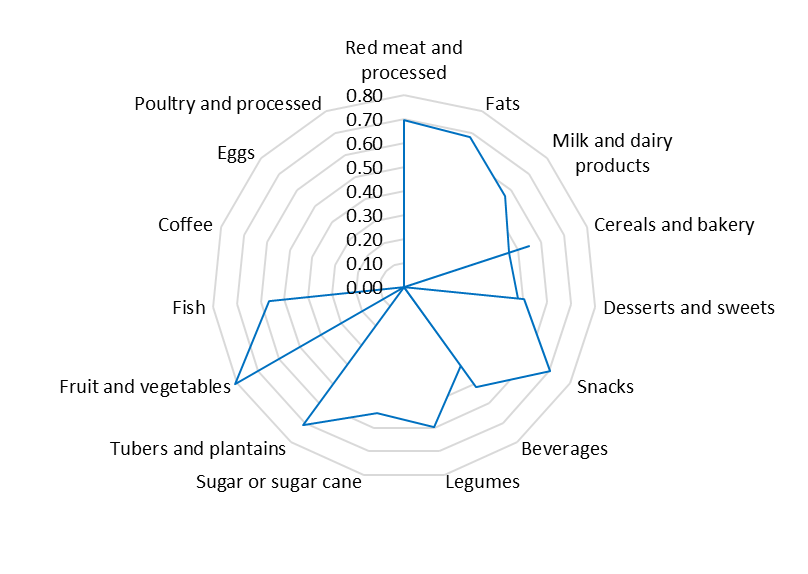 |
| D | Oriental |  |
|  | before | during |
|  | 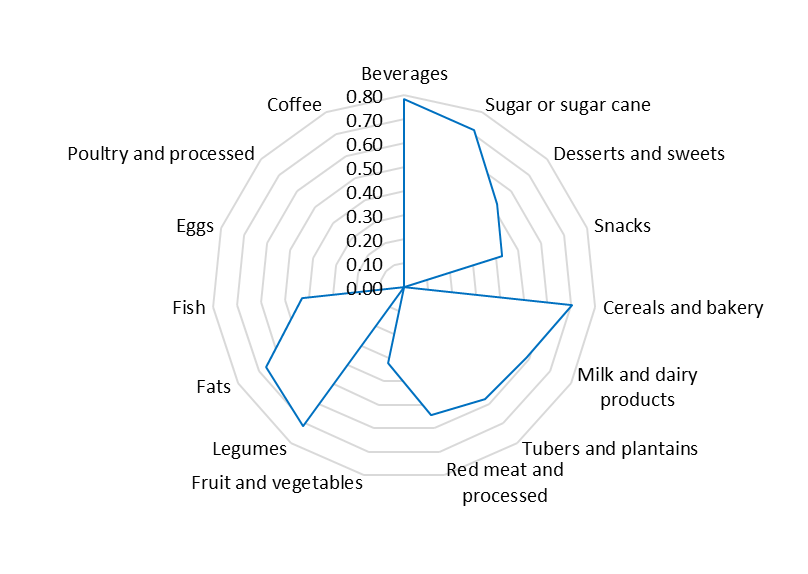 | 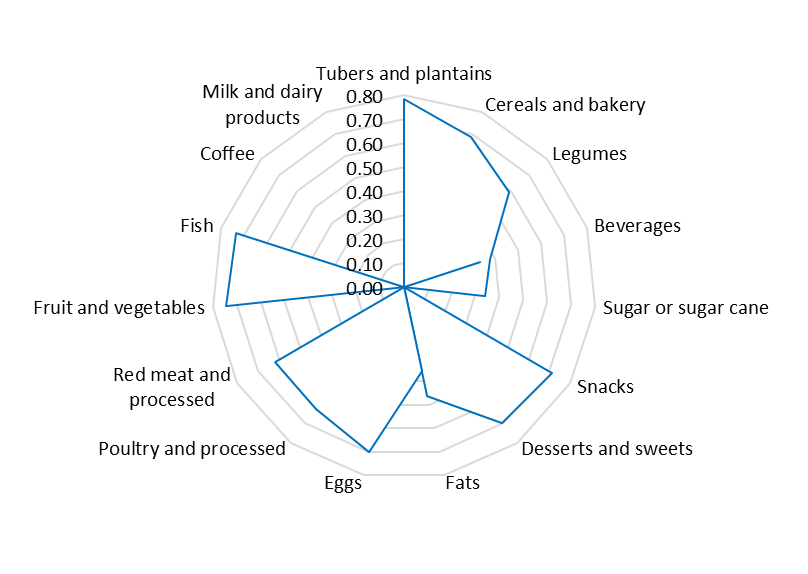 |
| E | Orinoquía and Amazonía |  |
|  | before | during |
|  | 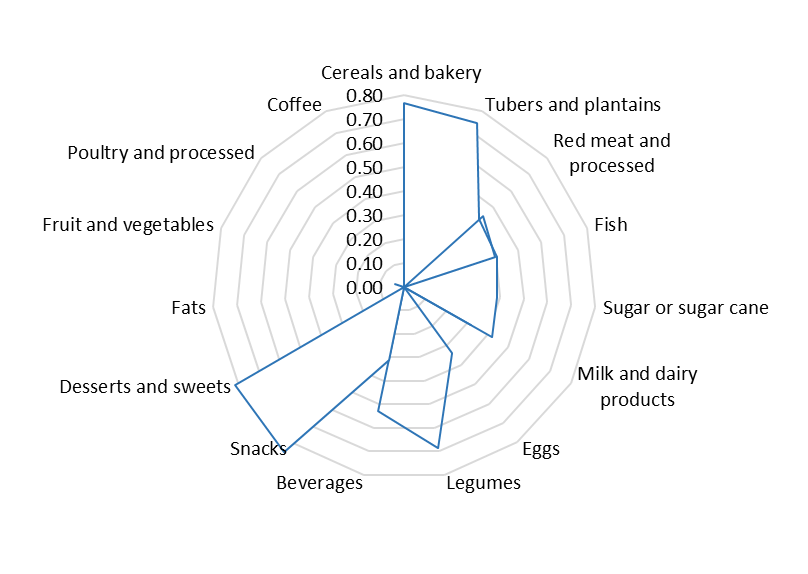 | 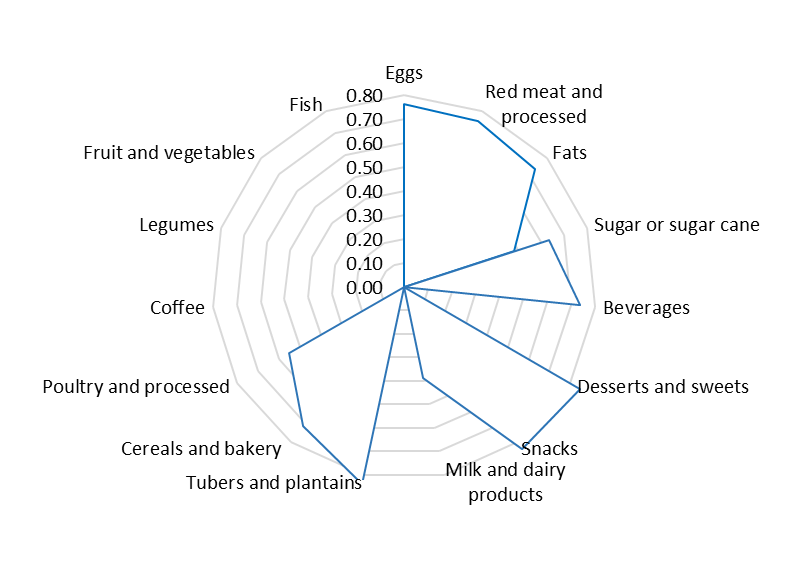 |
| F | Pacífica |  |
|  | before | during |
|  | 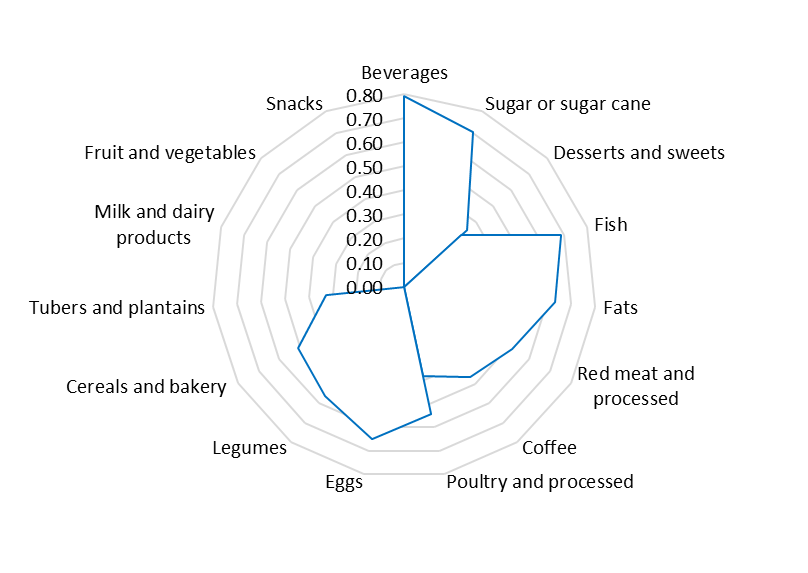 | 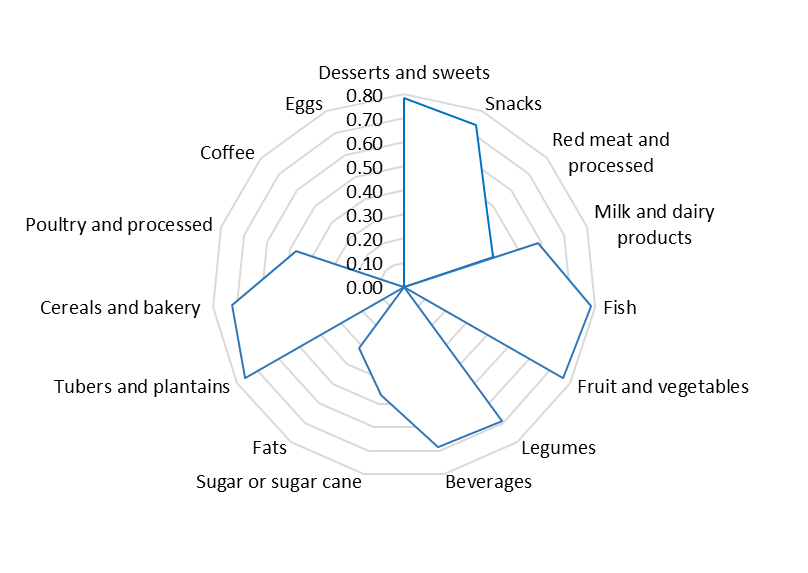 |

**Supplementary Figure 5.** Factor loadings of each food group and their contribution to the dietary patterns derived from principal component analysis for every region, before (A) and during (B) confinement.

**A-Before confinement**


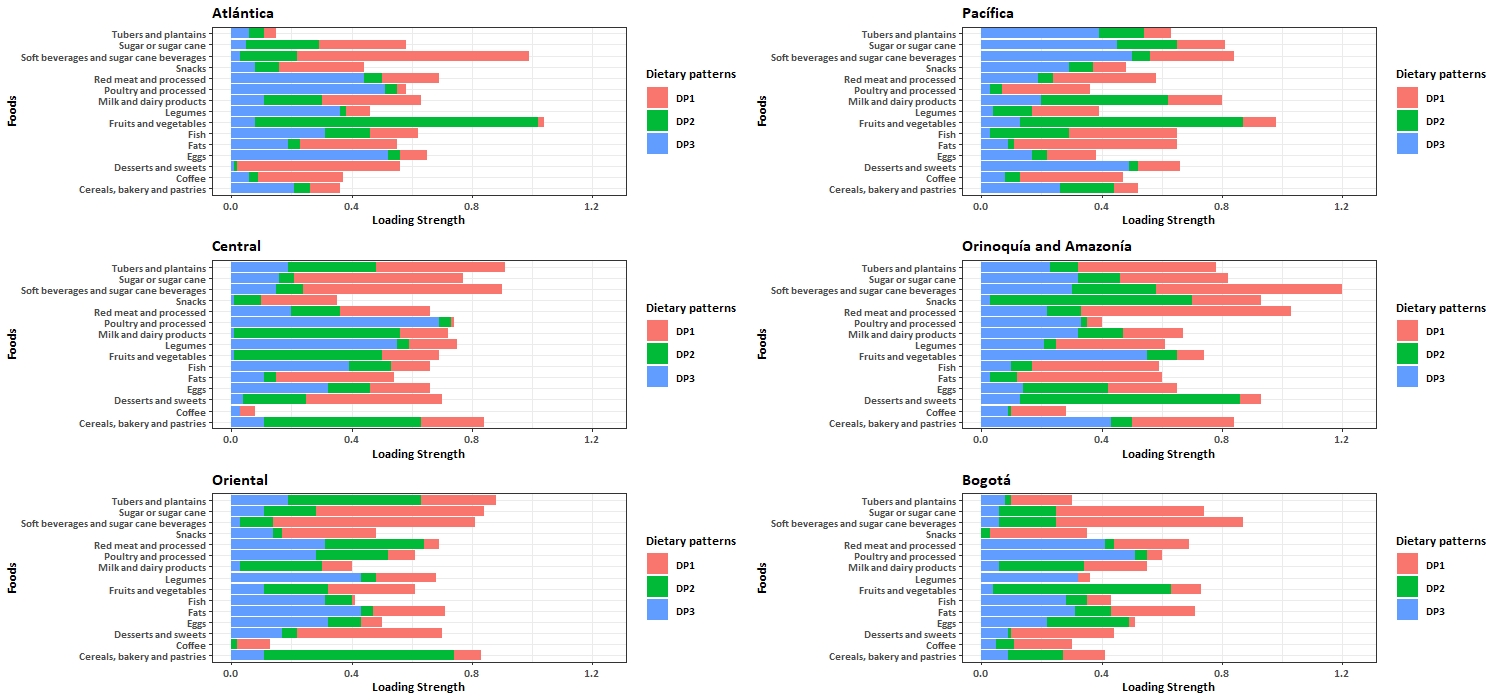


**B-During confinement**


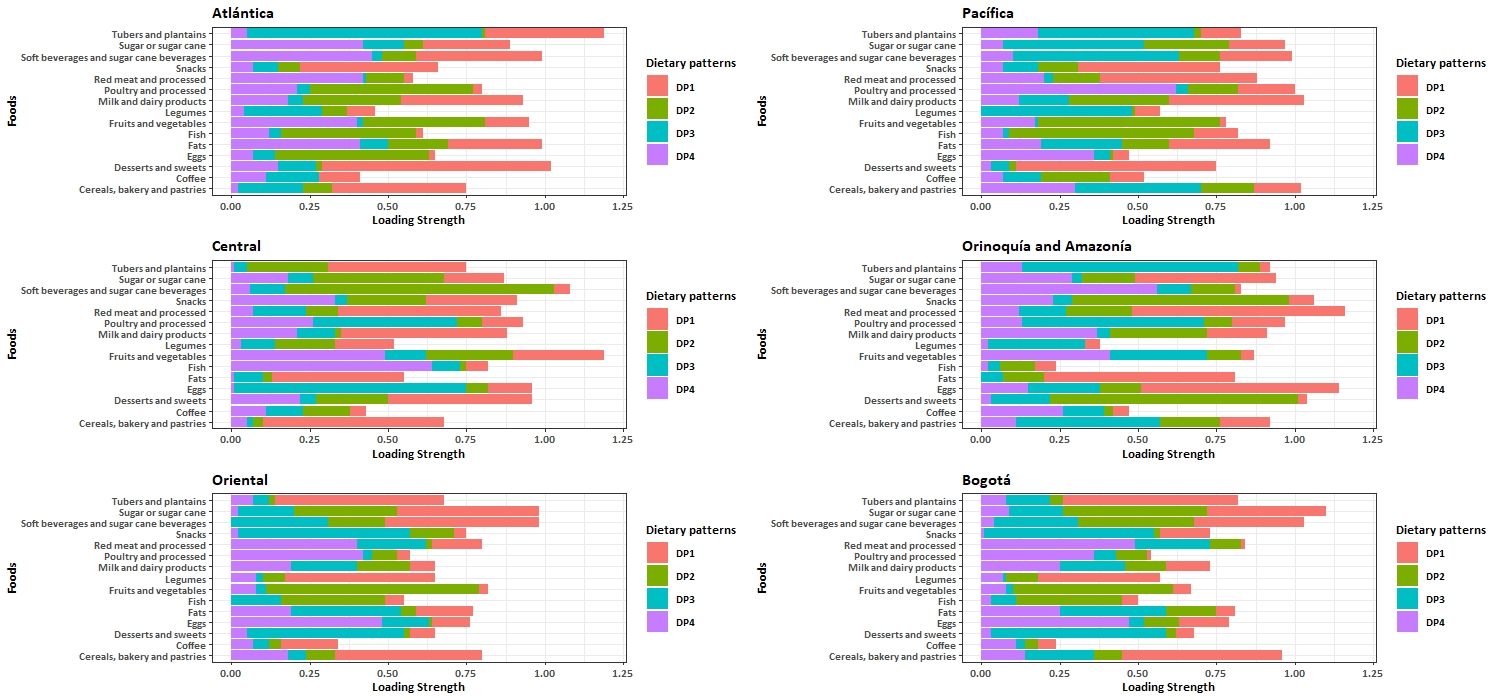


**
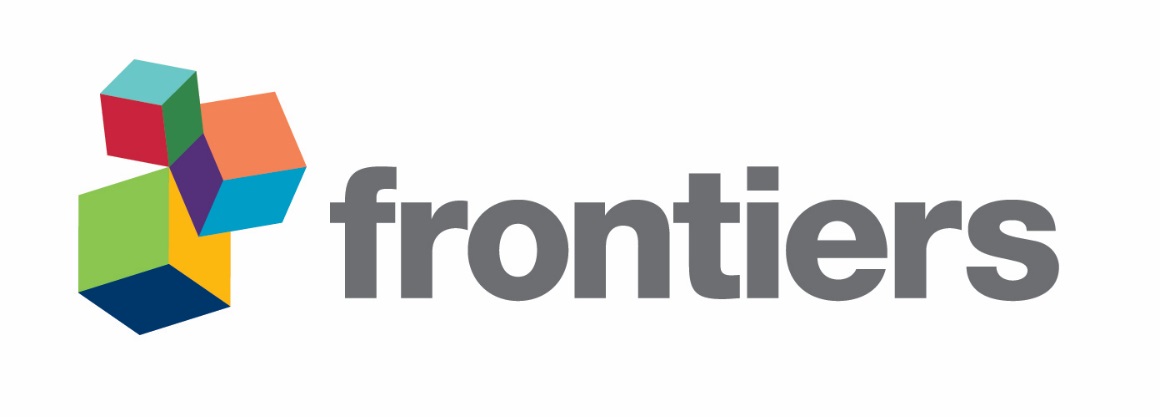
**
